# Lysosomal exoglycosidases as biomarkers of systemic diseases – what’s new?

**DOI:** 10.3389/fmolb.2026.1891977

**Published:** 2026-10-16

**Authors:** Iwona Ptaszyńska-Sarosiek, Weronika Wiktoria Sarosiek, Gabriela Iga Sarosiek, Anna Charuta, Mateusz Maciejczyk

**Affiliations:** 1 Department of Forensic Medicine, Medical University of Bialystok, Bialystok, Poland; 2 Independent Researcher, Białystok, Poland; 3 Institute of Health Sciences, Faculty of Medical and Health Sciences, University of Siedlce, Siedlce, Poland; 4 Department of Hygiene, Epidemiology and Environmental Health President of the Polish Hygiene Society, Bialystok Branch, Medical University of Bialystok, Bialystok, Poland

**Keywords:** biomarkers, glycoconjugate catabolism, glycosaminoglycan homeostasis, lysosomal enzymes, lysosomal exoglycosidases, systemic diseases

## Abstract

Numerous studies suggest that lysosomal exoglycosidases can be utilised in the diagnosis and monitoring of various diseases. This paper summarises recent reports on the clinical use of N-acetyl-β-D-hexosaminidase (HEX), β-glucuronidase (GLU), β-galactosidase (GAL), α-mannosidase (MAN) and α-L-fucosidase (FUC) in systemic diseases. The clinical relevance of exoglycosidases has been demonstrated in liver disease, alcohol use disorder (AUD) in adults, nicotine addiction, kidney disease, cardiovascular disease, diabetes, cancer, nasopharyngeal disease and musculoskeletal disorders. Exoglycosidase activity correlates with disease severity and is characterised by high sensitivity, though low specificity. However, lysosomal exoglycosidases may have a particular application in addiction medicine. Compared to routine biomarkers, they are more sensitive and specific to alcohol-induced damage. Importantly, lysosomal exoglycosidases (especially HEX and its isoenzymes) show a greater correlation with acute ethanol exposure and are less dependent on individual factors (age, gender, comorbidities).

## 1 Introduction

A biomarker is a characteristic that can be objectively measured to assess physiological and pathological processes occurring in a living organism, serving as a physiological, morphological, biochemical, or molecular indicator to assess health status, disease progression, or treatment efficacy (Aronson and Ferner, 2017; Biomarkers Definitions Working Group Biomarkers, 2001). The characteristics of an “ideal biomarker” include high sensitivity and specificity, the potential to differentiate pathologies at an early stage of the disease, a long half-life in a biological sample, as well as an easy, rapid and inexpensive method of measurement (Zweig and Campbell, 1993; Strimbu and Tavel, 2010). In epidemiology, biomarkers are generally classified into three categories: biomarkers of exposure, which detect foreign substances or their metabolites or products resulting from interactions between harmful agents and target cells or molecules; biomarkers of effect, which measure biochemical, physiological, behavioral, and other internal changes that may reflect existing health conditions or indicate potential future health issues; and biomarkers of susceptibility, which serve as indicators of either innate or acquired capacity to respond to exposure to specific toxicants (Traczewska, 2011).

Biomarkers can be sourced from various biological fluids, such as blood, urine, saliva, cerebrospinal fluid, or synovial fluid, as well as from tissues and organs (Aronson and Ferner, 2017; Chen et al., 2011). The vast majority of biomarkers are proteins, and it is not only the concentration but also the activity of the enzymatic protein that is diagnostically significant. Numerous studies suggest the promising potential of lysosomal enzymes as biomarkers in laboratory medicine (Paciotti et al., 2019; Ali et al., 2018; Olszewska-Slonina et al., 2015; Pagliardini et al., 2015; Benkoe et al., 2015; Parnetti et al., 2014). Lysosomes contain over 50 hydrolytic enzymes that can degrade all types of cellular macromolecules (Alberts et al., 2007; Cooper, 2000; Cuervo and Dice, 1997). Exoglycosidases such as N-acetyl-β-D-hexosaminidase (HEX), β-glucuronidase (GLU), β-galactosidase (GAL), α-mannosidase (MAN) and α-L-fucosidase (FUC) show particularly high activity among lysosomal enzymes (Chojnowska et al., 2017). Lysosomal exoglycosidases catalyse the hydrolysis of terminal glycosidic bonds. Therefore, the substrates for exoglycosidases are glycoproteins, glycolipids and glycosaminoglycans, and these reactions result in the trimming of the terminal monosaccharide residues (Zalewska-Szajda et al., 2019a). Since each monosaccharide residue is removed separately, a series of exoglycosidases is required, each of which hydrolyses a specific glycosidic bond. Exoglycosidases exhibit activity in an acidic environment, which provides a certain degree of protection for cytoplasmic components against autolysis (Cooper, 2000; Lübke et al., 2009; Schulze et al., 2009). In light of recent reports, exoglycosidases (in particular HEX) may be potential biomarkers for many systemic diseases, encompassing a broad clinical context, encompassing disorders associated with systemic metabolic, inflammatory, degenerative, or neoplastic processes. Despite major advances in laboratory diagnostics, many currently used biomarkers have important limitations. They demonstrate insufficient sensitivity for early disease detection, limited specificity due to overlap between different pathological conditions, and susceptibility to confounding factors such as age, sex, comorbidities, medications, and lifestyle factors (Biomarkers Definitions Working Group Biomarkers, 2001). Liver enzymes such as alanine aminotransferase (ALT) and aspartate aminotransferase (AST) reflect hepatocellular injury but do not reliably distinguish between different etiologies of liver disease (European Association for the Study of the Liver, 2021). Biomarkers used in nephrology, including serum creatinine and estimated glomerular filtration rate (eGFR), increase only after substantial loss of renal function (Stevens and Levin, 2013). Similarly, cardiovascular biomarkers may indicate established tissue injury rather than early pathological changes (Chow et al., 2017). Tumour markers frequently suffer from limited specificity and may be elevated in benign conditions (Duffy, 2013). Consequently, there is a growing interest in identifying novel biomarkers capable of detecting disease at earlier stages and providing complementary information regarding tissue remodeling and cellular damage.

This paper summarises the latest reports on the use of lysosomal exoglycosidases in the diagnosis of liver disease, alcohol use disorder, nicotine addiction, kidney disease, cardiovascular disease, diabetes, cancer, nasopharyngeal disease and musculoskeletal disorders. The present review does not attempt to cover all systemic disorders. The diseases discussed were selected because sufficient clinical evidence regarding lysosomal exoglycosidase activity is currently available (Platt et al., 2018). Although neurodegenerative disorders, respiratory diseases, and other chronic inflammatory conditions may also involve disturbances in glycoconjugate metabolism, clinical studies investigating exoglycosidases in these settings remain scarce (Nixon, 2013).

## 2 Structure and properties of lysosomal exoglycosidases

N-acetyl-β-D-hexosaminidase (HEX; EC 3.2.1.52) is the most active enzyme among lysosomal exoglycosidases (Vellodi, 2005). It is present in almost all human tissues and organs, especially in the liver, kidneys, gastric mucosa, spleen, brain, placenta, lungs, and skin fibroblasts (Zwierz et al., 1992). HEX consists of two subunits (α and β) (Zwierz et al., 1999). The α subunit is encoded by the *hexosaminidase subunit alpha (HEXA) gene* located on chromosome 15q23, while the β subunit is encoded by the *hexosaminidase subunit beta (HEXB) gene* located on 5q13.3 (Hexosaminidases) The three best-known HEX isoenzymes are HEX-A (αβ), HEX-B (ββ) and HEX-S (αα) (Zwierz et al., 1999). The other isoenzymes include pregnant HEX (HEX-P) (ββ), intermediate forms of HEX (HEX-I1 (probably ββ) and HEX-I2), as well as cytosolic forms of HEX of distinct genetic origin. The cytosolic forms of HEX include HEX-C (encoded by O-GlcNAcase [OGA] located on 10q24.32) and HEX-D (encoded by hexosaminidase D [HEXD] located on 17q25.3) (Zwierz et al., 1999; Hexosaminidases; Geiger et al., 1978; Pérez and Tutor, 1998; Isaksson et al., 1989; Tommasini et al., 1985; Gutternigg et al., 2009). Of all the isoenzymes, HEX-A and HEX-B emerge as the most dominant, occurring in varying proportions in different organs (Ostrowska et al., 1993). HEX-A is most commonly found in serum, urine and synovial fluid, while HEX-B is mainly present in the liver, placenta and kidneys. HEX catalyses the hydrolysis reaction of terminal non-reducing N-acetyl-D-hexosamine residues in N-acetyl-β-D-hexosaminides (N-acetylglucosides and N-acetylgalactosides) (Figure 1). (Ostrowska et al., 1993) Thus, the substrates for HEX are various sugar derivatives, the most important of which are GM_2_ gangliosides (Ostrowska et al., 1993). When HEX activity in nerve cells is reduced or absent, gangliosides accumulate, leading to a gradual decline in normal nervous system function (Ostrowska et al., 1993). Accumulation of GM_2_ gangliosides in brain cells is responsible for intellectual disability, motor disorders, blindness or loss of speech. Storage disorders associated with congenital HEX deficiency, such as Tay–Sachs disease (GM_2_ gangliosidosis) and Sandhoff’s disease, have a particularly severe clinical course. They are genetic and incurable diseases that cause death in childhood (Ostrowska et al., 1993). Until 5–6 months of age, infants with Tay–Sachs disease develop normally, but eventually present with visual and hearing disorders, mobility problems and intellectual disability (Vellodi, 2005). A significant decrease in HEX-A activity in serum, leucocytes and skin fibroblasts is key to diagnosing Tay–Sachs disease (Vellodi, 2005). Tay–Sachs disease can be diagnosed during pregnancy by assessing HEX-A activity in amniotic fluid (Zalewska-Szajda et al., 2019a). Deficiency of HEX-A and HEX-B activity causes Sandhoff’s disease, whose symptoms are similar to those of Tay–Sachs disease. Sandhoff’s disease is accompanied by the storage of oligosaccharides in internal organs, mainly in the liver and spleen (Vellodi, 2005). The symptoms of Sandhoff’s disease are non-specific and include muscle weakness, lack of coordination (ataxia), speech disorders and intellectual disability. To confirm Sandhoff’s disease diagnosis, HEX activity should be evaluated in serum, leucocytes or skin fibroblasts (Vellodi, 2005).

**FIGURE 1:**
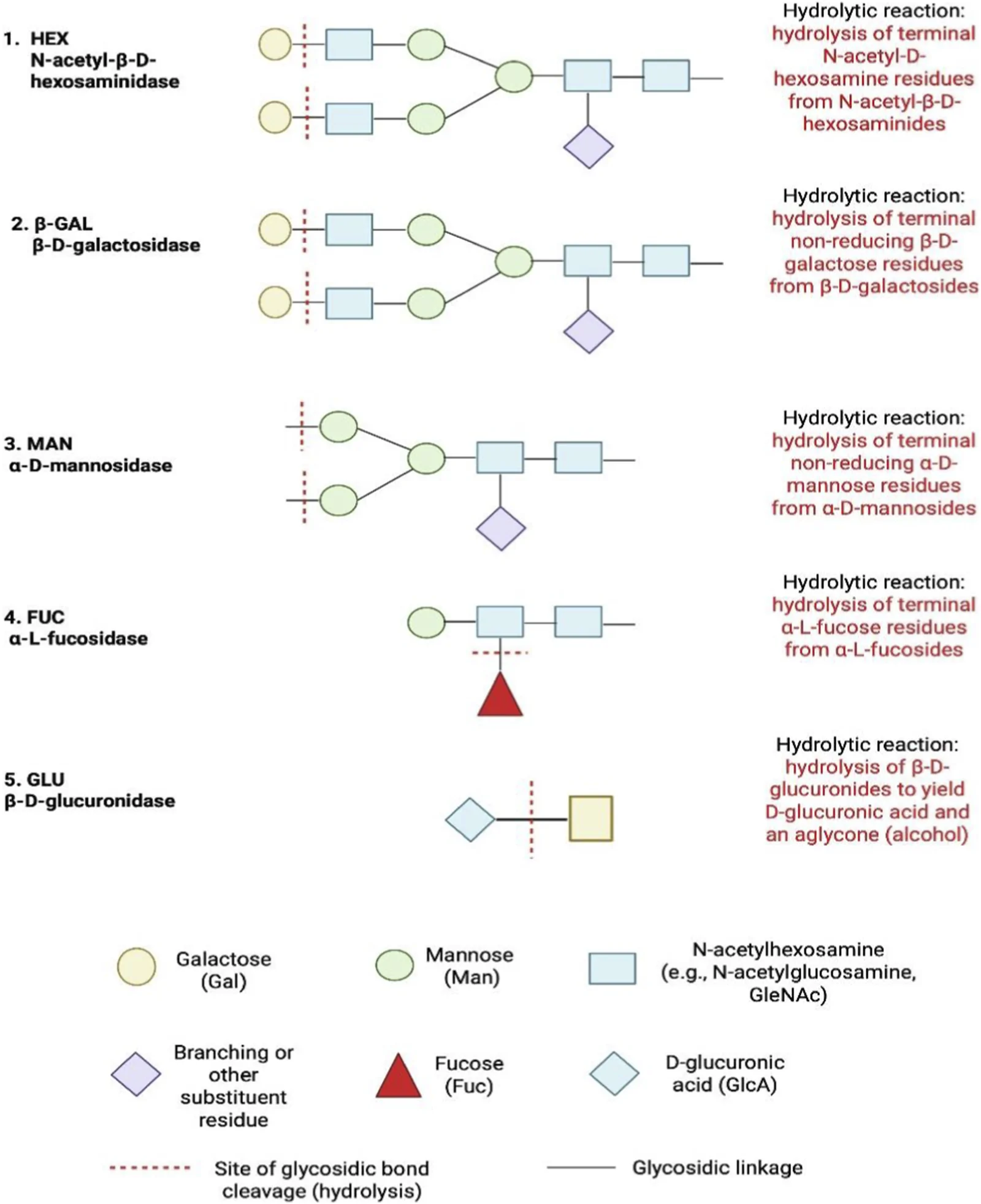
Hydrolytic activity of selected lysosomal exoglycosidases and schematic representation of their substrates and cleavage sites. The figure illustrates the action of lysosomal exoglycosidases involved in the stepwise degradation of glycoconjugates, including glycoproteins, glycolipids, and glycosaminoglycans. Each enzyme catalyses the hydrolysis of specific terminal, non-reducing monosaccharide residues from glycan chains, as indicated by the red dashed lines. Together, these enzymes act sequentially to remove individual monosaccharide residues, enabling the complete lysosomal degradation of complex glycoconjugates.

Alongside HEX, β-glucuronidase (β-glucuronide glucuronohydrolase glucuronidase; GLU; EC 3.2.1.31) also exhibits relatively high activity (Albandar et al., 1998). GLU is a tetrameric glycoprotein found in the human body in two forms: endogenous and exogenous (Albandar et al., 1998). Endogenous GLU is produced in the liver, kidneys, spleen, intestinal epithelium, salivary glands and the gonads (Albandar et al., 1998). The increase in GLU production and secretion is particularly evident during inflammation (Albandar et al., 1998). GLU is considered a marker of neutrophil influx and is released by primary granules (Albandar et al., 1998; Maciejczyk et al., 2017). Exogenous GLU, on the other hand, is produced by bacteria colonising the gut. The enzyme catalyses the reaction between β-D-glucuronoside and water, resulting in the formation of D-glucuronate and alcohol (Figure 1). GLU activity reflects the intensity of glycosaminoglycan catabolism (Buddecke and Gabius, 2009; Marciniak et al., 2006). Hereditary GLU deficiency causes a metabolic disorder called Sly’s syndrome (mucopolysaccharidosis type VII) (Vellodi, 2005). Sly’s syndrome can cause hydrops fetalis. If the child is born, it may have mental disorders, intellectual disability, growth deficiency, coarse facial features, and enlarged liver and spleen (Vellodi, 2005).

β-galactosidase (GAL; EC 3.2.1.23) is present in the small intestine, liver, kidneys, lungs and placenta (Czartoryska, 1977). There are three GAL isoenzymes: A, B and C, with GAL-C activity being low (Czartoryska, 1977). GAL catalyses the hydrolysis of terminal non-reducing β-D-galactose residues in β-D-galactosides (Figure 1). (Pérez and Tutor, 1998) GAL activity reflects the intensity of degradation of glycoproteins, glycolipids and glycosaminoglycans containing galactose (Czartoryska, 1977). Deficiencies in the enzyme can result in galactosialidosis or Morquio syndrome B (mucopolysaccharidosis type IVB) (Arbisser et al., 1977).

There are three isoenzymes of α-mannosidase (MAN; EC 3.2.1.24): A, B, and B2, which differ in sialic acid content and amino acid structure (Cheng et al., 1986). Indeed, MAN exists in the form of several polypeptides with molecular weights ranging from 40 to 67 kDA (Cheng et al., 1986). MAN catalyses the hydrolysis of terminal, non-reducing α-D-mannose residues in α-D-mannosides (Figure 1) (Malm and Nilssen, 2008) Moreover, MAN hydrolyses α-D-lyxosides and heptopyranosides with the same configuration at C-2, C-3 and C-4 as mannose. (Malm and Nilssen, 2008) MAN deficiency occurs in a storage disease called mannosidosis, marked by immunodeficiency, skeletal abnormalities, facial dysmorphism, and intellectual disability. (Malm and Nilssen, 2008)

In turn, α-L-fucosidase (FUC; EC 3.2.1.51) is an enzyme found in various tissues, including the liver, kidneys, lymphocytes, placenta, spleen, pancreas, brain, muscles and skin. There are two FUC isoenzymes: FUC I and FUC II, which differ in molecular weight (Thorpe and Robinson, 1975). FUC catalyses the reaction of an α-L-fucoside with water to produce an L-fucose and alcohol (Figure 1). The activity of lysosomal FUC reflects the intensity of degradation of glycoproteins containing α-L-fucose and glycolipids (Li et al., 2006; Bierc et al., 2010). The absence or deficiency of FUC activity results in fucosidosis. Fucosidosis is confirmed by a decrease in FUC activity in fibroblasts or leucocytes (Vellodi, 2005).

## 3 Pathophysiological basis of lysosomal exoglycosidases as biomarkers

Lysosomal exoglycosidases are predominantly intracellular enzymes localized within lysosomes (Settembre et al., 2013). Under physiological conditions, only small amounts are released into extracellular fluids. During tissue injury, inflammation, oxidative stress, ischemia, fibrosis, or accelerated cellular turnover, lysosomal membrane permeability increases, resulting in leakage of enzymes into the extracellular space and circulation. Activated macrophages, neutrophils, fibroblasts, and damaged parenchymal cells may additionally secrete lysosomal enzymes through regulated exocytosis (Andrews, 2000). Because glycoconjugates are major structural components of cell membranes, extracellular matrix proteins, and basement membranes, enhanced glycoconjugate degradation accompanies many pathological processes. Consequently, elevated exoglycosidase activity reflects intensified tissue remodeling and degradation occurring in multiple organs. This mechanism may explain why lysosomal exoglycosidases are altered in apparently unrelated diseases affecting the liver, kidneys, cardiovascular system, joints, and malignant tissues (Ballabio and Bonifacino, 2020). Figure 2 illustrates the adaptation from current concepts of lysosomal exocytosis, extracellular matrix remodeling, and glycoconjugate turnover (Settembre et al., 2013; Ballabio and Bonifacino, 2020; Theocharis et al., 2016).

**FIGURE 2:**
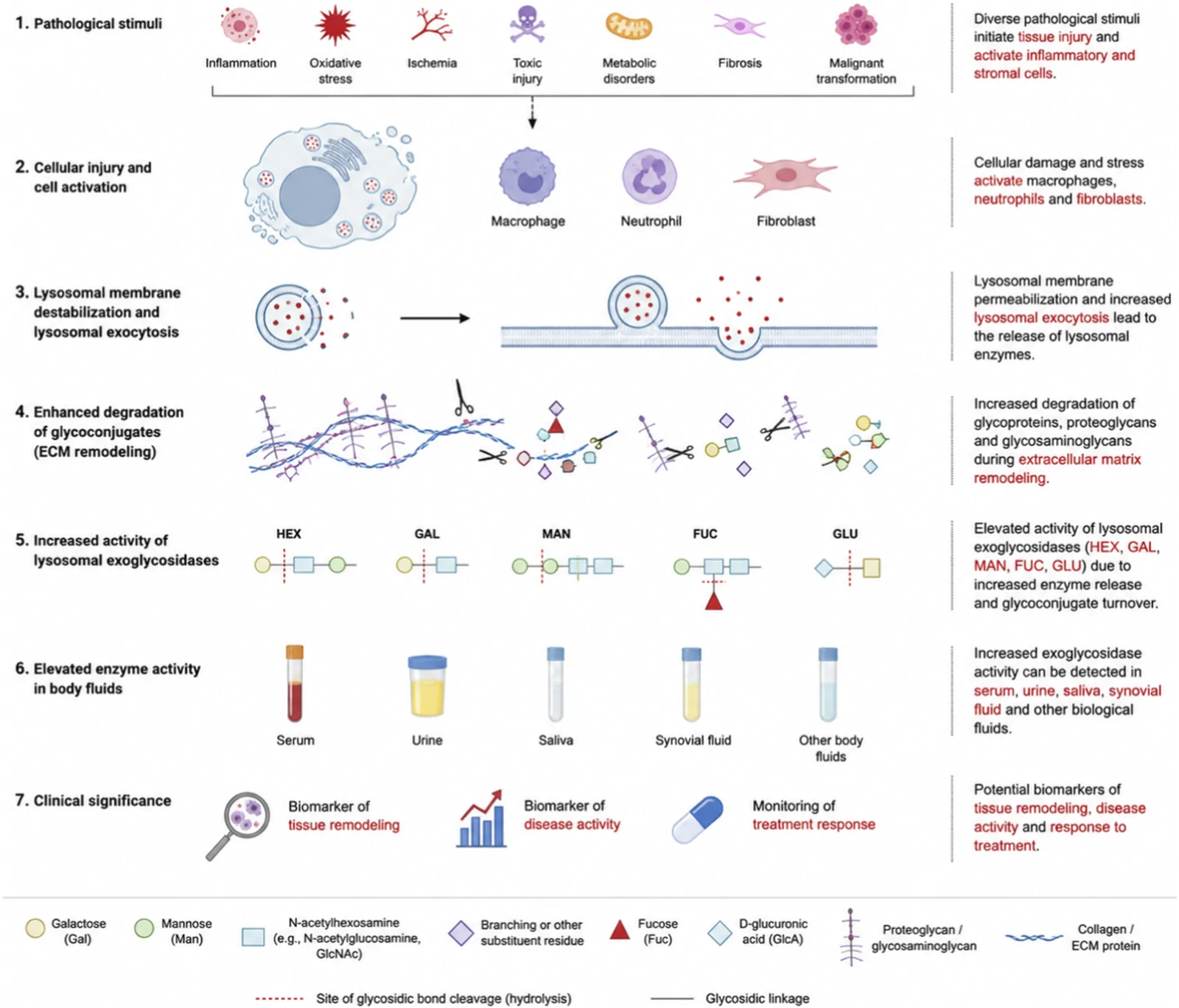
Pathophysiological mechanisms linking systemic diseases with increased lysosomal exoglycosidase activity. Diverse pathological stimuli, including inflammation, oxidative stress, metabolic disturbances, toxic injury, fibrosis, and malignant transformation, promote cellular damage and extracellular matrix remodeling (Theocharis et al., 2016). These processes increase glycoconjugate degradation and lysosomal enzyme release, leading to elevated exoglycosidase activity in biological fluids, where they may serve as biomarkers of disease activity and tissue remodeling (Iozzo and Schaefer, 2015).

## 4 Clinical significance of lysosomal exoglycosidases

Numerous studies suggest that lysosomal exoglycosidases can be utilised in the diagnosis and monitoring of various diseases (Chojnowska et al., 2017; Chojnowska et al., 2011). The clinical relevance of exoglycosidases has been demonstrated in liver disease, alcohol use disorder (AUD), nicotine addiction, kidney disease, cardiovascular disease, diabetes, cancer, nasopharyngeal disease and musculoskeletal disorders (Table 1; Supplementary Table S1).

**TABLE 1:** Clinical significance of lysosomal exoglycosidases.

Disease	Biomarker	Biological material	Results	References
Liver diseases				
Acute hepatitis	HEX	Serum	↑	(Hultberg et al., 1981)
Chronic hepatitis	GLU	Serum	↑	(Ohta, 1991)
Alcoholic liver disease	HEX	Serum	↑	(Faber et al., 1984)
	GLU			
	MAN			
	FUC			
	HEX	Serum	↑	(Reglero et al., 1980)
	FUC			
Diet-induced liver damage	HEX	Liver	↑	(Żebrowska-Gamdzyk et al., 2018)
	GLU			
	MAN			
	FUC			
Alcohol use disorder and substance-related disorders				
Alcohol use disorder	HEX	Serum	↑	(Waszkiewicz et al., 2008)
	HEX-A	Serum		
	HEX-B	Serum		
	HEX	Saliva		
	HEX-A	Urine		
	HEX-A	Saliva	↑	(Waszkiewicz et al., 2019)
	GLU	Saliva		
	FUC	Saliva		
	MAN	Saliva		
	HEX-B	Serum	↑	(Hultberg et al., 1991)
	HEX-A	Saliva	↑	(Waszkiewicz et al., 2013)
	HEX-B	Saliva	↓	
	HEX	Serum	↑	(Ptaszyńska-Sarosiek et al., 2020)
	HEX-A			
	HEX-B			
	HEX	Urine/CSF/vitreoss humour	↓	
	HEX-A			
	HEX-B			
	HEX	Urine	↑	(Taracha et al., 1999)
Nicotine addiction	HEX	Urine	↑	(Nowak et al., 2014)
	HEX	Urine	↑	(Buchet et al., 1990)
Kidney diseases				
Ureteropelvic junction obstruction	HEX	Urine	↑	(Ellis et al., 1975)
	HEX-A			
	HEX-B			
	GLU			
	FUC			
	MAN			
	GAL			
Tubular injury in patients a solitary functioning kidney	HEX	Urine	↑	(Taranta‐Janusz et al., 2015)
	HEX-A			
	HEX-B			
	GLU			
	FUC			
	MAN			
	GAL			
Hydronephrosis	HEX	Urine	↑	(Ellis et al., 1975)
	HEX-A			
	HEX-B			
	GLU			
	FUC			
	MAN			
	GAL			
Renal tumours	HEX	Urine	↑	(Sanna-Cherchi et al., 2009)
Kidney transplantation	HEX	Urine	↓	(Liubimova et al., 1997b)
	HEX-B	Urine	≈	
Diabetic nephropathy	HEX	Urine	↑	(Hultberg et al., 1990)
Cardiovascular diseases				
Hypertension	HEX-A	Urine	↑	(Matuszak et al., 2010)
	HEX	Urine	↑	(Benito et al., 2024)
	HEX-A			
	HEX-B			
	HEX	Urine	↑	(Chobanian et al., 2003)
Atherosclerosis	HEX	Serum	↑	(Beręsewicz and Skierczyńska, 2006)
	HEX-A			
	HEX-B			
	HEX	Serum	↑	(Hultberg et al., 1994)
	HEX-A			
	HEX-B			
Metabolic diseases				
Diabetes	HEX	Saliva	↑	(Brownlee, 2005)
	GLU	Serum	↑	(Severini et al., 1988)
	HEX	Serum	↑	(Zalewska-Sza et al., 2013)
		Urine		
Cancers				
Colorectal cancer	GLU	Serum	↑	(Cox and Erler, 2011)
	HEX	Serum/urine	↑	(Waszkiewicz et al., 2015)
	HEX-A			
	HEX-B			
	HEX	Serum/urine	↑	(Pancewicz et al., 2009)
	GAL	Serum/urine	↑	
	FUC	Serum	↑	
	MAN	Serum	↑	
	FUC	Urine	≈	
	MAN	Urine	≈	
Liver cancer	FUC	Serum	↑	(Giardina et al., 1998)
	FUC	Plasma	↑	(Gan et al., 2014)
Pancreatic cancer	HEX	Serum	↑	(Szajda et al., 2008a)
	HEX-A	Serum	↑	
	HEX-B	Serum/urine	≈	
Kidney cancer	HEX	Serum/urine	↑	(Popko et al., 2006)
	HEX	Renal tumour tissue	↓	(Wang et al., 2014)
	HEX-A			
	HEX	Transitional tissue	≈	
	HEX-A			
Thyroid cancer	HEX-A	Serum	↑	(Borzym-Kluczyk et al., 2006)
	HEX-B	Serum	↓	
Brain tumours	HEX	Brain glioma tissues	↑	(Waszkiewicz et al., 2009b)
	HEX-A			
	HEX-B			
	GAL			
	MAN			
Salivary gland tumours	HEX	Tissue of pleomorphic adenoma	↑	(Wielgat et al., 2006)
	HEX-A			
	HEX-B			
	HEX	Salivary gland tumour tissue	↑	
	HEX-A			
	HEX-B			
	GAL			
	FUC			
	HEX	Serum	↑	
	HEX-A			
	HEX-B			
	GLU			
	GAL			
	MAN			
	FUC			
	HEX	Saliva	↑	
	HEX-A	Saliva	↑	
	HEX-B	Saliva	↓	
	GLU	Saliva	↑	
	GAL	Saliva	↑	
	MAN	Saliva	↑	
	FUC	Saliva	↑	
Laryngeal cancer	MAN	Laryngeal cancer	↑	(Borzym-Kluczyk et al., 2008)
	GAL			
Other tumours	HEX	Serum	↑	(Olszewska et al., 2012)
	GLU			
Nasopharyngeal diseases				
Nasal polyps	GAL	Nasal polyp tissue	↓	(Morimoto et al., 1971)
	FUC			
Chronic tonsillitis	HEX	Tonsil tissue	↑	(Zhang et al., 2003)
	HEX-A			
	HEX-B			
	GLU			
	GAL			
	MAN			
	FUC			
Rheumatic and musculoskeletal diseases				
Inflammatory joint diseases	HEX	Serum	↑	(Baszczuk and Kopczyński)
	GAL			
	MAN			
Systemic sclerosis	HEX	Saliva	↑	(Volkmann et al., 2023)
	HEX-A			
	HEX-B			
	GLU			

### 4.1 Liver diseases

Most studies on the diagnostic usefulness of exoglycosidases concern liver diseases. This fact should come as no surprise, since the liver is the main site of synthesis of these enzymes (Żebrowska-Gamdzyk et al., 2018). Conventional biomarkers of liver injury, including alanine aminotransferase (ALT), aspartate aminotransferase (AST), gamma-glutamyl transferase (GGT), and bilirubin, are widely used in clinical practice. However, these markers primarily reflect hepatocellular damage and often show limited ability to distinguish between different etiologies of liver disease or to detect early fibrotic changes (European Association for the Study of the Liver, 2021). Furthermore, their activity may be influenced by age, sex, obesity, medications, and AUD. Consequently, additional biomarkers reflecting extracellular matrix remodeling and glycoconjugate turnover may provide complementary diagnostic information (Castera et al., 2019; Giannini et al., 2005).

#### 4.1.1 Acute hepatitis

Acute hepatitis involves the rapid development of inflammatory and necrotic lesions. The most common cause of the disease are primary hepatotropic viruses (i.e., hepatitis A (HAV), B (HBV), C (HCV), hepatitis D virus (HDV) and E (HEV)) or secondary hepatotropic viruses (i.e., Epstein–Barr virus (EBV), cytomegalovirus (CMV), herpes simplex virus, varicella zoster virus (VZV) and adenoviruses) (Suriawinata and Thung, 2006). Hultberg et al. found that lysosomal exoglycosidases can be utilised in the diagnosis of acute hepatitis (Hultberg et al., 1981). Serum HEX activity was significantly elevated in acute hepatitis A and slowly decreased with treatment. Positive correlations were found between serum HEX activity and ALT activity (r = 0.44, p < 0.01) and AST activity (r = 0.40, p < 0.02). The changes in serum HEX activity in acute viral hepatitis B were similar. Positive correlations were found between serum HEX activity and ALT activity (r = 0.33, p < 0.05), AST activity (r = 0.46, p < 0.005), and alkaline phosphatase activity (ALP; r = 0.4, p < 0.02), as well as bilirubin concentration (r = 0.61, p < 0.001) (Hultberg et al., 1981).

#### 4.1.2 Chronic hepatitis

Chronic hepatitis is a liver inflammation lasting longer than 6 months as a result of necrotic and inflammatory changes (Walewska-Zielecka, 2000). The disease can be viral, drug-induced, autoimmune, alcohol-related, or associated with fatty liver disease (Weber et al., 2011). Chronic hepatitis is often asymptomatic and is only detected by specialised imaging studies. Therefore, new biomarkers are constantly sought to detect the disease before serious complications can arise. Ohta et al. indicate that serum GLU activity was significantly higher in patients with liver cancer, cirrhosis and chronic active hepatitis compared to the control group (Ohta, 1991). A positive correlation was found between GLU activity and the degree of liver cell necrosis determined by histological observation. It was confirmed that necrotic liver cells are the source of GLU in serum. Eight out of ten patients with labile and elevated serum GLU activity showed progression of chronic hepatitis. It is suggested that GLU activity measurement may be used to predict the progression of hepatitis (Ohta, 1991).

#### 4.1.3 Alcoholic liver disease

Liver damage in alcoholic liver disease develops in stages: starting with alcoholic fatty liver disease, through alcoholic hepatitis, and ending with alcoholic cirrhosis. However, the individual stages of alcoholic liver disease are not clearly differentiated from one another, which makes proper treatment difficult (Mackowiak et al., 2024). Therefore, it is essential to develop biomarkers that indicate the clinical stage of the disease. Faber et al. assessed the activity of lysosomal exoglycosidases in the serum of patients with alcoholic liver disease confirmed by biopsy or a history of chronic AUD (Faber et al., 1984). The authors reported a significant increase in the activity of HEX (1.4-fold), GLU (2.1-fold), acid MAN (2.2-fold), intermediate MAN (1.6-fold) and FUC (2.3-fold) in the serum of patients with liver disease compared to the control group. MAN activity was significantly higher (p < 0.001) when measured in an acidic environment compared to measurements at neutral pH. Reglero et al. suggest that cirrhosis alters lysosome integrity and membrane permeability (Reglero et al., 1980). Authors indicated significantly higher FUC and HEX activity in serum in patients with diabetes, cirrhosis and gastric cancer compared to the control group, and no significant differences in the activity of other glycosidases. Marshall et al. (1974) demonstrated that serum from patients with alcoholic cirrhosis and liver metastases may contain elevated levels of desialylated glycoproteins, thus providing evidence of abnormalities in the carbohydrate recognition system. In patients with mechanical jaundice, the levels of these glycoproteins were similar to those of healthy individuals.

#### 4.1.4 Diet-induced liver damage

Żebrowska-Gamdzyk et al. demonstrated that a high-protein diet based on whey proteins may impair liver function. The authors investigated the effect of whey protein concentrate (WPC-80) on glycoconjugate catabolism, as well as selected markers of oxidative stress and liver inflammation. In liver homogenates, the authors examined the activity of HEX, GLU, GAL, MAN, FUC, as well as the levels of reduced glutathione (GSH), malondialdehyde (MDA), interleukin-1β (IL-1β) and transforming growth factor-β1 (TGF-β1). Significantly higher HEX, GLU, MAN and FUC activity was found in the livers of rats receiving WPC-80 compared to the controls. Serum alanine aminotransferase (ALT) and aspartate aminotransferase (AST) concentrations were significantly higher in animals supplemented with WPC-80 at 0.5 g/kg body weight for 21 days. Increased levels of GSH, MDA, IL-1β and TGF-β1 were also observed in the same group of animals. WPC-80 contributes to intense liver tissue remodelling and induces oxidative stress, particularly at a dose of 0.5 g/kg body weight (Żebrowska-Gamdzyk et al., 2018).

### 4.2 Alcohol use disorder and substance-related disorders

#### 4.2.1 Alcohol use disorder

According to the WHO, more than two billion people worldwide consume alcohol (https://www.who.int) Alcohol is ranked third and, in Europe, second among population health risk factors. Excessive AUD remains a major public health problem and is associated with substantial morbidity and mortality worldwide. Early identification of individuals engaging in harmful drinking patterns is important for both prevention and treatment. (https://www.who.int) Biomarkers currently used to identify excessive AUD, such as GGT, carbohydrate-deficient transferrin (CDT), mean corpuscular volume (MCV), and phosphatidylethanol (PEth), differ in sensitivity, specificity, and detection window. None of these markers alone reliably identifies all individuals (AUD) or heavy alcohol consumption. Therefore, additional biomarkers capable of reflecting alcohol-induced tissue damage may improve diagnostic accuracy (Helander and Hansson, 2013; Stewart et al., 2014). In recent years, increasing attention has been directed toward lysosomal exoglycosidases, particularly (HEX), as potential biomarkers of excessive AUD and alcohol-related tissue injury.

Numerous studies report that HEX and HEX-B are sensitive and specific markers of AUD (Conigrave et al., 2003; Cylwik et al., 2007; KDIGO (n. d.); Neuman et al., 2003; Niemelä, 2007; Waszkiewicz et al., 2010a; Waszkiewicz et al., 2010b; Waszkiewicz et al., 2011; Winchester, 2005). Increased HEX activity in serum and urine was found in alcohol-dependent patients. The term “alcohol-dependent patients” refers to individuals who exhibit impaired control over alcohol intake, tolerance, withdrawal symptoms, and continued alcohol use despite adverse health or social consequences. According to current DSM-5 terminology, such individuals are classified as having Alcohol Use Disorder (AUD). HEX activity also increases in healthy individuals after consuming more than 60 g of alcohol per day for at least 10 consecutive days (Sharpe, 2001). It was also observed that even a single high dose of ethanol can increase the activity of total β-HEX, β-HEX A, and β-HEX B in serum, as well as total β-HEX in saliva and β-HEX A in urine (Waszkiewicz et al., 2008). Acute alcohol intake is the consumption of alcohol during a single drinking occasion or over a short period of time, leading to immediate but generally transient physiological and biochemical effects. Acute alcohol intake (6 h) of a high but tolerable dose of alcohol (120–160 g) significantly increases the activity of total HEX and the HEX-A and HEX-B isoenzymes in serum, as well as total HEX in saliva and HEX-A in urine in infrequent drinkers (Waszkiewicz et al., 2008). It was shown that the increase in total HEX activity in serum and urine is mainly caused by the secretory isoenzyme HEX-A (Waszkiewicz et al., 2008). In alcohol-dependent individuals, HEX activity returns to normal after 7–10 days of sobriety in serum and urine, and after 4 weeks in saliva (Waszkiewicz et al., 2011; Waszkiewicz et al., 2019). Hultberg et al. analysed HEX isoenzymes in the serum of people with (AUD) hospitalised for detoxification compared to abstinent people with AUD and a reference group of non-drinkers. Serum HEX-B was significantly elevated in all 38 patients hospitalised for detoxification. In the 38 abstainers, only one had elevated levels of HEX-A, HEX-B, and total HEX compared to the control group. HEX-B had a half-life of 2–4 days in hospitalised patients, while the half-lives of HEX-A and gamma-glutamyltransferase (GT) differed significantly. No clear pattern of decrease in HEX-A and GT was found, as in the case of HEX-B. HEX-B correlated significantly with total HEX and HEX-A, while HEX-A correlated with HEX, AST and GT (r = 0.99, p < 0.001). HEX-B was found to be a useful marker of AUD (Hultberg et al., 1991). Waszkiewicz et al. also examined HEX-A% (percentage of β-HEX A isoenzyme to total β-HEX) and HEX-B% (percentage of β-HEX B isoenzyme to total β-HEX) in the saliva of people with AUD with periodontal disease. HEX-A% was significantly higher and HEX-B% lower in the alcohol-dependent smoker group compared to the healthy group. The high AUC value for *β*-HEX A% (0.937) in alcohol-dependent smokers, excellent sensitivity (96%) and specificity (91%), and its correlation with the gingival index (GI) indicate the high value of *β*-HEX A% as an excellent marker of periodontal disease (Waszkiewicz et al., 2013).

Diagnostic usefulness in assessing AUD is demonstrated not only by HEX and its isoenzymes but also by other exoglycosidases. Waszkiewicz et al. report that exoglycosidase activity in saliva was 2–3 times (MAN), 2–6 times (FUC), 8–25 times (HEX-A) and 19–40 times (GLU) higher in groups of alcohol-dependent non-smokers and alcohol-dependent smokers than in controls (healthy individuals who drink socially) and was characterised by very high sensitivity, specificity and accuracy (Waszkiewicz et al., 2019). Numerous correlations were also reported between the duration of alcohol use disorder and the activity of GLU, HEX-A, FUC and MAN in saliva, as well as between the amount of alcohol consumed and the activity of HEX-A in saliva. HEX activity in saliva was significantly higher in alcohol-dependent non-smokers and smokers compared to non-drinkers and non-smokers. On day 15 of abstinence, significantly lower FUC, MAN and GAL activity were observed in alcohol-dependent non-smokers compared to alcohol-dependent smokers. The authors suggest that HEX-A in saliva may be a potential marker of chronic alcohol intake, FUC and MAN as markers of AUD, and GLU as a marker of chronic alcohol intake and alcohol use disorder (Sharpe, 2001). The literature outlines various mechanisms by which HEX activity increases in circulating body fluids after alcohol intake. These include changes in lysosomal membrane permeability and enzyme leakage from liver lysosomes, followed by the release of enzymes from hepatocytes into body fluids, delayed enzyme removal by the damaged liver, and increased enzyme synthesis through the activation of reticuloendothelial cells (Waszkiewicz et al., 2009a). Since deglycosylation of glycoconjugates can lead to proteolysis, it can be speculated that high HEX activity in alcohol-dependent individuals may affect the activity of other markers of AUD (AST, ALT and GT).

Ptaszyńska-Sarosiek et al. assessed the activity of HEX, HEX-A and HEX-B in blood, urine, cerebrospinal fluid and vitreous humour in alcohol poisoning fatalities (alcohol concentration of 4‰ and above) compared to sudden death cases. The highest serum HEX activity was found in both the study and control groups. Significantly lower HEX, HEX-A and HEX-B activity was observed in the urine of alcohol poisoning victims compared to those who died sober (Ptaszyńska-Sarosiek et al., 2020). HEX and its isoenzymes A and B were higher only in blood serum in both study groups, and this may be related to liver damage. In ethanol poisoning fatalities, lower HEX, HEX-A, and HEX-B activity was observed in urine, cerebrospinal fluid, and vitreous humour compared to blood serum, which may be evidence of blood–organ barrier damage. Decreased HEX activity in urine can be explained by harmful alcohol use rather than AUD. In alcohol poisoning fatalities, significantly lower levels of HEX, HEX-A and HEX-B in urine than in blood serum may indicate significant kidney damage caused by ethanol and its metabolites, particularly acetaldehyde. Studies revealed the highest HEX-A activity in the urine of fatal ethanol poisoning victims compared to HEX and HEX-B, which may be explained by ethanol-induced inflammatory kidney damage. The decrease in HEX activity and its isoenzymes in urine can also be explained by lower vasopressin (antidiuretic hormone) secretion in people who consume large amounts of ethanol (Ptaszyńska-Sarosiek et al., 2020).

Taracha et al. investigated the usefulness of measuring HEX activity in the urine of patients undergoing methadone substitution treatment to identify cases of AUD (Taracha et al., 1999). It was found that methadone, illicit drugs and HIV seropositivity did not affect the activity of this enzyme. However, β-HEX activity was significantly higher in patients who admitted to occasional heavy drinking compared to those who declared abstinence (p = 0.014) and compared to healthy controls (p = 5 × 10^−8^). Heavy drinking refers to alcohol intake exceeding recommended low-risk drinking limits and is associated with an increased risk of adverse health outcomes (National Institute on Alcohol Abuse a nd Alcoholism) β-HEX activity was still lower than in detoxified alcohol-dependent subjects (p = 0.024). Given that HEX sensitivity in differentiating people with AUD was 87% and specificity was 80%, measuring HEX activity in urine may help assess the effects of AUD treatment (Taracha et al., 1999).

In conclusion, serum HEX-B activity, total HEX in urine, and HEX-A in saliva may be highly sensitive biomarkers of AUD (Waszkiewicz et al., 2010b; Sharpe, 2001; Waszkiewicz et al., 2018). HEX activity increases in the urine and blood of alcohol-dependent individuals and those who consume more than 60 g per day for at least 10 days (Sharpe, 2001), returning to normal after 7–10 days of sobriety in serum and urine and after 4 weeks in saliva (Waszkiewicz et al., 2011; Waszkiewicz et al., 2019). Despite numerous reports on the diagnostic potential of lysosomal exoglycosidases in alcoholic disease, validation of biomarkers in a larger patient population is necessary.

### 4.2.2 Nicotine addiction

Smoking exposure is commonly assessed using biomarkers such as cotinine, exhaled carbon monoxide, and nicotine metabolites (Benowitz et al., 2009). Although these markers are highly useful for confirming recent tobacco exposure, they do not provide information regarding smoking-induced tissue injury or early organ damage (Chang, 2009). Consequently, biomarkers reflecting the biological effects of tobacco smoke, including renal and vascular injury, may offer complementary diagnostic value (U.S. Department of Health and Human Services, 2014).

The WHO estimates that there are more than 1.1 billion smokers worldwide (https://www.who.int) The medical consequences of smoking include an increased risk of cardiovascular disease, malignant tumours, respiratory and kidney disease, as well as infertility and pregnancy complications (Nowak et al., 2014). Hultberg et al. found that urinary HEX excretion in 203 apparently healthy men and women correlated significantly with tobacco consumption (r = 0.26, p < 0.001), whereas urinary creatinine excretion did not differ between smokers and non-smokers (Hultberg et al., 1992). Individuals who smoked more than 10 g of tobacco per day excreted significantly more HEX in urine than non-smokers, which may indicate the presence of low-grade kidney damage in smokers (Hultberg et al., 1992). One reason for the increase in exoglycosidase activity in the urine of smokers may be smoking-related cadmium exposure (Buchet et al., 1990). Cadmium is a poison that accumulates in tissues, mainly in the kidneys, and has a biological half-life of more than 10 years. Histopathologically, there is an increase in the size and number of lysosomes in renal tubular cells of smokers (Kawada et al., 1989). Another reason for increased exoglycosidase activity in smokers may be smoking-related hypoxia, which leads to increased HEX excretion by tubular cells. Nicotine also enhances the release of lysosomal enzymes from macrophages (Schwartz and Bond, 1972) and increases glomerular filtration rate (Pawlik et al., 1985).

### 4.3 Kidney diseases

Renal function is routinely assessed using serum creatinine, estimated glomerular filtration rate (eGFR), and albuminuria () However, these parameters may remain within normal ranges during the early stages of kidney injury and often reflect established rather than incipient damage (Waikar and Bonventre, 2009). Biomarkers indicating tubular dysfunction and extracellular matrix remodeling are therefore being actively investigated as tools for earlier detection of renal pathology (Ostermann et al., 2020).

Kidney disease is the third fastest growing cause of death worldwide. By 2040, it will become the fifth most common cause of death (Time to sound the alarm, 2024). Most kidney diseases are asymptomatic and often only diagnosed at an advanced stage of development. This accelerates the need to start renal replacement therapy (Król and Rutkowski, 2008). Therefore, it is necessary to search for sensitive, non-invasive, and inexpensive methods for assessing kidney function and early diagnosis of renal diseases (Benito et al., 2024).

Urinalysis is one of the basic laboratory tests to assess renal function (Zalewska-Szajda et al., 2019b; Abirami and Tiwan, 2001). Lysosomal exoglycosidases are among the few enzymes whose activity can also be determined in urine (Abirami and Tiwan, 2001; Kiliś-Pietrusińska and Zwolińska, 1999). Exoglycosidase activity in urine increases significantly in many kidney diseases, such as acute and chronic glomerulonephritis (Dance et al., 1970), uraemia (Linko-Löppönen, 1986), vesicoureteric reflux (Kunin et al., 1978), chronic pyelonephritis (Filutkiewicz, 1979), nephrotic syndrome (Ellis et al., 1975), renal tubular injury in children with ureteropelvic junction obstruction (Taranta‐Janusz et al., 2015) and in children with a solitary kidney (Taranta-Janusz et al., 2014), after trauma and kidney transplantation (Bugge et al., 1999). Importantly, exoglycosidases are not denatured in the acidic pH of urine (Marchewka et al., 2014). In contrast, their activity is age-dependent. Zalewska-Szajda et al. (2018a) showed an inverse correlation between age and the activity of HEX and its isoenzymes in urine per creatinine: HEX: r = −0.24, p < 0.001; HEX A: r = −0.20, p < 0.01 and HEX B: r = −0.26, p < 0.001. The findings are partly consistent with Agirbasli et al. (Agirbasli et al., 1996), who demonstrated that HEX activity in the urine of young people (aged 18–32) depends on age, sex, exercise and blood pressure. The authors also noted a negative correlation between urinary HEX, HEX-A and HEX-B activity (pKat/μg Cr) and age in a study of children and adolescents with a solitary functioning kidney and hydronephrosis (Taranta‐Janusz et al., 2015; Taranta-Janusz et al., 2014). Zalewska-Szajda et al. (2018b) found that the activity of FUC, GAL, GLU and MAN in the urine of 203 children and adolescents, as in the case of HEX and its isoenzymes (Zalewska-Szajda et al., 2018a), is independent of sex, but depends on age and is statistically significantly higher (p < 0.001) (pKat/μg Cr) in the urine of the youngest children (0.5–2.9 years) compared to adolescents (15.0–17.9 years). Furthermore, the authors demonstrated a negative correlation between the age of the children and adolescents and the activity in urine (pKat/μg Cr): FUC (r = −0.36, p < 0.0001); GAL (r = −0.36, p < 0.0001); GLU (r = −0.35, p < 0.0001) and MAN (r = −0.35, p < 0.0001) (Marchewka et al., 2014). The relationship between the activity of lysosomal exoglycosidases in the urine of children and adolescents and age and centile range within the normal values of the third, 10th, 25th, 50th, 75th, 90th and 97th centiles may be of practical importance in differentiating between healthy children and adolescents and those who are ill (Zalewska-Szajda et al., 2018a; Zalewska-Szajda et al., 2018b).

#### 4.3.1 Ureteropelvic junction obstruction

The consequence of ureteral stricture is obstructed or absent urine flow from the kidney (Zalewska-Szajda et al., 2019b). This leads to a series of changes at the cellular level, including apoptosis and mesenchymal–myocyte transformation, which is manifested by progressive damage to the glomeruli and renal tubules (Chevalier et al., 2002). These processes result in the release of HEX, among other substances. Taranta-Janusz et al. found that the activity of lysosomal exoglycosidases (HEX, HEX-A, HEX-B, FUC, GAL, MAN, and GLU) in urine was significantly higher in children with ureteropelvic junction obstruction than in healthy children (Taranta‐Janusz et al., 2015).

#### 4.3.2 Tubular injury in patients with a solitary functioning kidney

The prevalence of solitary functioning kidney (SFK) is one in 2,000 individuals (Westland et al., 2013). Approximately 40%–50% of adults with SFK require dialysis (Sanna-Cherchi et al., 2009). Lysosomal enzymes may be useful biomarkers of tubular injury in children with a solitary functioning kidney (Taranta-Janusz et al., 2014). The activity of HEX, HEX-A, HEX-B, FUC, GAL, MAN, and GLU in urine was significantly higher in children with tubular injury and a solitary functioning kidney (Taranta-Janusz et al., 2014) than in healthy patients (p < 0.05). Compared to the control group, patients with congenital solitary functioning kidney (cSFK) showed significantly higher activity for all exoglycosidases (p < 0.05). Similar results were observed in children with acquired solitary functioning kidney (aSFK), excluding HEX-A (p > 0.05). Very strong positive correlations were found between the activity of all urinary exoglycosidases and the albumin/creatinine ratio (p < 0.001). The median estimated glomerular filtration rate (eGFR) correlated negatively with age in patients with SFK (r = −0.33, p < 0.01). A negative correlation was demonstrated between HEX, its isoenzymes HEX-A and HEX-B (pKat/mL), and eGFR (r = −0.29, r = −0.25, r = −0.29, p < 0.05, respectively) in children with SFK. These findings may indicate damage to the renal tubules in children with a solitary functioning kidney, with high and low glomerular filtration rates.

#### 4.3.3 Hydronephrosis

Taranta-Janusz et al. demonstrated that the activity of HEX, HEX-A, HEX-B, FUC, GAL, MAN and GLU in urine was significantly higher in children with hydronephrosis caused by ureteropelvic junction obstruction than in healthy children (Taranta‐Janusz et al., 2015).

#### 4.3.4 Renal tumours

Liubimova et al. investigated the usefulness of assessing exoglycosidase activity in urine to predict nephrotoxicity in 19 children with malignant solid tumours who received polychemotherapy with cisplatin at 100 mg/m^2^ or ifosfamide at 9 g/m^2^ (Liubimova et al., 1997a). After chemotherapy, hyperenzymuria persisted, with significantly higher levels of HEX, GTP and alanine aminopeptidases (ANAases) in the urine compared to baseline levels. Treatment with fosfamide significantly increased HEX activity in urine compared to patients treated with cisplatin (Liubimova et al., 1997a). Liubimova et al. concluded that HEX is a more sensitive marker of renal tubular injury caused by cytostatic drugs than urea and the albumin/creatinine ratio (Liubimova et al., 1997a; Liubimova et al., 1997b).

#### 4.3.5 Kidney transplantation

Hultberg et al. studied HEX concentration and HEX-B percentage using the enzyme immunoassay (EIA) method in the urine of kidney transplant patients treated with cyclosporine A, prednisolone and azathioprine. In patients after a kidney transplant without complications, a significant decrease (approaching a normal level) in HEX activity in urine and no change in the percentage of HEX-B were observed (Hultberg et al., 1990). In contrast, increased HEX activity in urine is an early indicator of kidney transplant rejection (Hultberg et al., 1990).

#### 4.3.6 Diabetic nephropathy

It was reported that HEX activity in the urine of children in the early stages of diabetic nephropathy is twice as high as HEX activity in the urine of healthy children (Romero et al., 1993).

### 4.4 Cardiovascular disease

Current cardiovascular biomarkers, including troponins, natriuretic peptides, and inflammatory markers, are valuable in diagnosing specific clinical conditions (Braunwald, 2008). Nevertheless, many of these markers reflect advanced tissue injury or systemic inflammation and provide limited information regarding ongoing vascular remodeling (Libby et al., 2009; López et al., 2015). Identification of biomarkers associated with glycoconjugate degradation may contribute additional insight into disease progression.

#### 4.4.1 Hypertension

Hypertension is the most common cardiovascular disease (Matuszak et al., 2010). The prevalence of hypertension increases with age, rising sharply after 50. The vast majority (more than 90%) of hypertension cases are primary (idiopathic). However, the aetiology of hypertension has not been fully identified (Matuszak et al., 2010). Genetic and environmental factors are thought to play an important role in it. Recent studies indicate that chronic inflammation and oxidative stress contribute to the induction of hypertension (Zalewska-Szajda et al., 2019c; Trott and Harrison, 2014). Inflammatory processes and oxidative stress are associated with tissue degradation and an increase in the concentration of various proteins in tissues and body fluids, including cell adhesion molecules, cytokines, acute-phase proteins (Winchester, 2005; Baszczuk and Kopczyński) and lysosomal enzymes (Pancewicz et al., 2009; Szajda et al., 2008a; Szajda et al., 2008b; Waszkiewicz et al., 2014; Szajda et al., 2010; Popko et al., 2006; Borzym-Kluczyk et al., 2005; Gallavan et al., 1984; Chobanian et al., 2003). Zalewska-Szajda et al. (2019c) reported that HEX-A activity in the urine of children with prehypertension (when systolic blood pressure is between 120 and 139 mm Hg and/or diastolic blood pressure is between 80 and 89 mm Hg) (Zalewska-Szajda et al., 2019c) was significantly higher than in children with normal blood pressure. Furthermore, the group of children with prehypertension showed a positive correlation between HEX and HEX-A and systolic blood pressure (r = 0.17, p < 0.05; r = 0.21, p < 0.05, respectively). The AUC differentiating these two groups was 0.616 for HEX-A (cut-off value of 29.351 pKat/mL; sensitivity 51.2%, specificity 71.8%) and 0.589 for HEX (cut-off value of 0.054 pKat/μgCr; sensitivity 31.7%, specificity 86.3%) (Zalewska-Szajda et al., 2019c). Other studies also indicated (Zalewska-Szajda et al., 2019b; Zalewska-Szajda et al., 2019c) that HEX-A can be a valuable tool for identifying children and adolescents with elevated blood pressure. The authors found a significant positive correlation between HEX-A activity and systolic blood pressure in boys, as well as a significant positive correlation between HEX, HEX-B, FUC, GAL, GLU and MAN activity and systolic blood pressure in girls (p < 0.05). Alderman et al. (1983) indicated that determining lysosomal exoglycosidase activity in the urine of hypertensive patients may be a useful marker of kidney injury. Studies by other authors (Lisowska-Myjak et al., 2011) suggested that urinary HEX activity may be an independent marker applicable to the diagnosis of early renal failure in hypertensive patients.

#### 4.4.2 Atherosclerosis

Atherosclerosis is a chronic inflammatory process affecting the aorta and medium-sized arteries (coronary, carotid, renal, and iliac arteries) (Beręsewicz and Skierczyńska, 2006). From a histological point of view, the process involves the accumulation of deposits in the space between the endothelium and the muscular layer of the vessel (intima), consisting only of macrophages, low-density lipoproteins (LDL), foam cells (macrophages loaded with oxidised LDL) and extracellular cholesterol clusters (Beręsewicz and Skierczyńska, 2006).

Hultberg et al. investigated serum levels of HEX isoenzymes in relation to risk factors for atherosclerosis in a homogeneous population of postmenopausal women (Hultberg et al., 1994). They found HEX to be associated with several risk factors, such as serum triglycerides, diastolic and systolic blood pressure, glycaemia, waist-to-hip ratio, and body mass index, but not with serum cholesterol. Furthermore, the mean values of HEX-A and HEX-B activity were higher in smokers than in non-smokers, but only the mean activity for HEX-A differed significantly. In addition, the authors demonstrated a positive correlation between serum HEX and risk factors for myocardial infarction and stroke. In the case of HEX-A, individuals in the 95th–100th percentile of enzyme activity showed a significantly increased risk of myocardial infarction if their fathers had this condition, as well as an increased likelihood of stroke if their mother had it. In the case of HEX-B, individuals in the 95th–100th percentile of enzyme activity showed an increased incidence of stroke if their mothers had a stroke. These studies suggest an association between HEX isoenzymes and the heritability of vascular disease. Since HEX-B is a sensitive marker of AUD, the authors also examined its serum levels in individuals suspected of AUD. However, the study found no differences in these individuals compared to others, probably due to the relatively short half-life of HEX-B after quitting drinking (Hultberg et al., 1994).

Chojnowska et al. (2018) analysed correlations between type 2 diabetes (T2D) and serum HEX activity with fat metabolism parameters and symptoms of anxiety and depression. The study was conducted on a random sample of patients with T2D aged 43–71 years (median 59) and controls aged 18–64 years (median 46). In patients with T2D, a significant positive correlation was found between serum HEX activity and serum concentrations of LDL cholesterol, triacylglycerols (TAGs) and Castelligro atherogenic indexes. A significantly increased level of anxiety and depression was also observed in comparison with the control group. The authors concluded that serum HEX activity in patients with T2D is a better marker of atherosclerosis than total serum cholesterol concentration in individuals with mild symptoms of depression and anxiety. Routine screening for anxiety and depression is recommended in patients with T2D. Early detection of these disorders provides an opportunity for treatment, improving the quality of life and overall life expectancy of patients with T2D.

### 4.5 Diabetes

Diabetes represents a group of metabolic disorders of various aetiology characterised by hyperglycaemia resulting from a defect in insulin secretion and/or action (Chojnowski et al.). Chronic hyperglycaemia contributes to the failure of various organs, mainly the heart, blood vessels, eyes, kidneys and nerves (Raport Komisji Ekspertów na temat Rozpoznawania i Klasyfikacji Cukrzycy, 2002). Diabetes has two primary forms: type 1 diabetes (autoimmune destruction of pancreatic β cells) and type 2 diabetes (progressive loss of the pancreatic β cells’ capacity to secrete insulin, accompanied by insulin resistance). Glycemic control is commonly assessed using fasting plasma glucose and glycated hemoglobin (HbA1c) (American Diabetes Association) Although these parameters are indispensable in clinical practice, they do not directly reflect tissue remodeling, extracellular matrix alterations, or the development of diabetic complications (Sherwani et al., 2016).Additional biomarkers may therefore improve early identification of organ damage associated with diabetes (Forbes and Cooper, 2013).Diabetes mellitus is associated with profound alterations in glycoconjugate metabolism (Brownlee, 2005).

Zalewska-Szajda et al. demonstrated that HEX activity in the saliva of children with type 1 diabetes was significantly higher compared to HEX activity in the saliva of healthy children (p < 0.001). In the group of children with diabetes, HEX activity in saliva was from ∼112.19 to ∼1,264.79 pKat/mL, average 494.33 ± 237.26 pKat/mL; specific HEX activity: from ∼0.1360 to ∼1.7526 pKat/µg protein, average 0.6266 ± 0.3561 pKat/µg protein. In the control group, HEX activity was from ∼126.12 to ∼398.40 pKat/mL, average 287.06 ± 111.34 pKat/mL; specific activity was from ∼0.1113 to ∼0.9908 pKat/µg protein, average 0.40340 ± 0.1978 pKat/µg protein. The differences between the groups were highly statistically significant: HEX activity concentration: p < 0.00001, specific activity: p < 0.001. HEX activity in saliva may be used to diagnose children with type 1 diabetes after validating the results on a larger population of children with diabetes (Zalewska-Sza et al., 2013). Severini et al. also showed significantly higher HEX activity in the serum and urine of diabetic patients (Severini et al., 1988). Total HEX activity was significantly increased (p < 0.001) in the serum and urine of patients with diabetes and vascular complications, but not in those with diabetes without vascular complications. The proportion of isoenzyme A activity always exceeded that of isoenzyme B.

Miller et al. report that GLU activity is significantly higher in the serum of patients with type 1 diabetes compared to the control group. The average enzyme activity was 2,160 units, while in the control group it was 1,260 units. Increased serum β-glucuronidase activity may be associated with pathological processes occurring in patients with type 1 diabetes. These results suggest that β-glucuronidase activity may serve as an indicator of disease activity or the extent of tissue damage in diabetes. This can be explained by the intensification of the glucuronic acid cycle, i.e., the glucose metabolism pathway that is insensitive to insulin. This pathway is more active in diabetes, stimulating the synthesis of mucopolysaccharides and glycoproteins, which results in increased GLU activity (Miller et al., 1966).

In patients with type 1 diabetes treated with insulin, a positive correlation was found between plasma HEX activity and blood glucose concentration (Poon et al., 1983). HEX activity also showed little diurnal variability, with the lowest level at 7.00 a.m. (p < 0.001). Mean plasma glucose and HEX levels fluctuated similarly (r = 0.88, p < 0.01). HEX activity correlated with changes in plasma glucose and glycated haemoglobin levels (r = 0.61, p < 0.025; r = 0.48, p < 0.05, respectively).

### 4.6 Cancers

In 2022, nearly 20 million new cases of cancer were diagnosed worldwide, of which 9.7 million patients died (Bray et al., 2024). The underlying cause of cancer is gene mutations. These mutations may emerge spontaneously as a result of errors in DNA replication or be caused by external factors such as UV radiation, tobacco smoke, chemicals or oncogenic viruses (Hahn and Weinberg, 2002). There are many types of genes whose mutations can lead to cancer, but the key ones are proto-oncogenes, tumour suppressor genes and DNA repair genes. Cancer develops when mutations accumulate in a way that prevents cells from controlling their growth, differentiation and apoptosis (Hahn and Weinberg, 2002)

Most currently used tumour markers, including carcinoembryonic antigen (CEA), cancer antigen 19-9 (CA19-9), prostate-specific antigen (PSA), and cancer antigen 125 (CA125), demonstrate limited sensitivity and specificity, particularly in early-stage disease (Duffy, 2007).

Moreover, elevated concentrations may occur in various non-malignant conditions (Hay et al., 2013). Consequently, there is ongoing interest in identifying additional biomarkers reflecting tumour-associated tissue remodeling and extracellular matrix degradation (Lu et al., 2012; Cox and Erler, 2011).

#### 4.6.1 Colorectal cancer

The existing literature indicates the usefulness of lysosomal exoglycosidase testing mainly in the diagnosis of gastrointestinal tumours. Waszkiewicz et al. reported that GLU activity in the serum of patients with colorectal adenocarcinoma is significantly higher compared to healthy controls (p < 0.0001). Serum GLU activity showed high sensitivity (80%) and specificity (82.35%) at a diagnostic threshold of >208.10 pKat/mL. AUC of the diagnostic test was 0.8956 (95% CI: 0.798–0.993), indicating a good potential to differentiate patients with colorectal cancer from healthy individuals (Waszkiewicz et al., 2015).

Szajda et al. examined the activity of HEX and its isoenzymes in the serum and urine of patients with colorectal adenocarcinoma, mucinous colorectal adenocarcinoma and healthy individuals. A significant increase in HEX, HEX-A and HEX-B activity was demonstrated in the serum and urine of patients with colorectal adenocarcinoma compared to healthy individuals. Patients with mucinous colorectal adenocarcinoma presented significantly higher serum HEX activity and significantly higher urine HEX and HEX-B activity expressed as pKat/mg creatinine compared to healthy individuals. A significant increase in HEX, HEX-A and HEX-B activity expressed in pKat/mg creatinine was observed in the urine of patients with tumours measuring 6–7 cm in diameter compared to patients with tumours of 4–5 cm in diameter. Determining HEX, HEX-A and HEX-B activity in blood serum and urine can be used to detect early-stage colorectal cancer.

Another study by Szajda et al. (2010) also showed a significant increase in HEX, GAL and FUC activity in the serum and HEX and GAL activity in the urine of patients with colorectal adenocarcinoma compared to healthy individuals. This implies that the detection of HEX and its isoenzymes in body fluids could be used to develop screening biomarkers for colorectal cancer detection.

#### 4.6.2 Liver cancer

Lysosomal exoglycosidases may also prove helpful in the diagnosis of liver cancer. Hepatocellular carcinoma (HCC) is a malignant tumour of the liver and the third leading cause of death worldwide due to late diagnosis. Serum FUC assessment appears to be a promising biomarker for detecting even small HCC tumours (Takahashi et al., 1994; Giardina et al., 1998). Gan et al. revealed that the sensitivity of FUC in diagnosing HCC is 0.72 (95% confidence interval (CI) of 0.69–0.76), with a specificity of 0.78 (95% CI of 0.74–0.81), a diagnostic odds ratio (DOR) of 10.26 (95% CI of 5.99–17.59) and an area under the curve (AUC) of 0.8125 (Gan et al., 2014). In addition, Wang et al. (2014) evaluated the prognostic value of preoperative serum FUC levels in patients with intrahepatic cholangiocarcinoma after liver resection. The authors report that preoperatively high serum FUC activity is a negative predictor of HCC. It was demonstrated that patients with preoperative plasma FUC above 35 uL^-1^ had a significantly lower recurrence-free survival, lower survival rate, and a greater tendency to develop macrovascular invasion.

#### 4.6.3 Pancreatic cancer

Adenocarcinoma is the most common malignant tumour of the pancreas (Waszkiewicz et al., 2009a). Biochemical diagnosis of pancreatic adenocarcinoma is based on the determination of tumour antigen (CA 19-9) in the blood, which is not, however, specific to this type of cancer (Waszkiewicz et al., 2009a). Therefore, non-invasive biomarkers for pancreatic cancer are still being sought. Szajda et al. (2008b) investigated HEX and its isoenzymes (HEX-A and HEX-B) in serum and urine as potential markers of pancreatic cancer. HEX and HEX-A specific activity was significantly higher in the serum of patients with pancreatic adenocarcinoma compared to healthy individuals. They found significantly higher HEX activity in the serum and urine of patients with pancreatic cancer compared to controls. GAL activity was also higher in the serum and urine of patients with pancreatic cancer compared to the control group. FUC and MAN activity were elevated in the serum, but no significant differences were found in the urine of patients with pancreatic cancer compared to controls. However, HEX-B-specific activity in serum and urine did not differ significantly between patients with pancreatic adenocarcinoma and controls (Szajda et al., 2008b). The results suggest that the activity of HEX and its isoenzyme A, as determined in serum and urine, can be used as a potential marker for pancreatic adenocarcinoma.

#### 4.6.4 Kidney cancer

Altered activity of lysosomal exoglycosidases was also reported in tumours involving other organs. The activity of HEX and its isoenzyme HEX-A was significantly lower in kidney cancer tissue compared to healthy tissue, while in transitional tissue, the enzyme activity was moderate. At the same time, a significant increase in HEX activity was observed in the serum and urine of patients with kidney cancer compared to healthy individuals (Borzym-Kluczyk et al., 2005). These observations confirm that exglycosidases are typical intracellular enzymes released as a result of cell breakdown and cell membrane damage. In cancerous and normal kidney tissues, the HEX-A isoenzyme showed statistically higher specific activity compared to the HEX-B isoenzyme (Borzym-Kluczyk et al., 2006).

#### 4.6.5 Thyroid cancer

It is proposed that lysosomal exoglycosidases could also be utilised in the diagnosis of thyroid cancer. Zwierz et al. (2006) assessed the concentration of thyroid-stimulating hormone (TSH) and the activity of HEX and its isoenzymes A and B in the blood serum of patients with thyroid cancer. The authors found a significant increase in the activity (p < 0.0191) and specific activity (p < 0.0393) of HEX-A, as well as a decrease in the specific activity of HEX-B, in the serum of patients with thyroid cancer. Normal TSH levels in the blood serum of patients with thyroid cancer confirm euthyroidism in the preoperative period. Determination of the activity and specific activity of HEX-A indicates that, despite euthyroidism, proteins indicative of a pathological process are released into the bloodstream in increased amounts. According to the authors, assessing TSH may help confirm euthyroidism in patients with thyroid cancer before surgery, while determining HEX-A activity may help diagnose thyroid cancer.

#### 4.6.6 Brain tumours

There is considerable evidence suggesting that modifications of cellular glycoconjugates may be important in tumour progression (Waszkiewicz et al., 2009b). Wielgat et al. found increased activity of HEX, HEX-A, HEX-B, GAL and MAN in brain glioma tissues compared to healthy brain tissue and non-glioma tumour tissue (Wielgat et al., 2006). The highest exoglycosidase activity was observed in highly malignant gliomas, specifically glioblastoma multiforme (grade IV), anaplastic astrocytoma (grade III), and anaplastic oligodendroglioma (grade III). HEX and HEX-B activity was six times higher than in normal brain tissue. Exoglycosidase activity in infiltrating tumours was significantly higher than in solid tumours. Furthermore, a positive correlation was observed between the activity of HEX, HEX-A and HEX-B and the malignancy grade of primary brain tumours (Wielgat et al., 2006). These results suggest that lysosomal exoglycosidases may be involved in the progression and dynamic development of gliomas.

#### 4.6.7 Salivary gland tumours

Changes in exoglycosidase activity were also noted in salivary gland tumours. Borzym-Kluczyk et al. report that the activity of HEX and its isoenzymes HEX-A and HEX-B in the tissue of pleomorphic adenoma, a benign tumour of the salivary gland, is significantly elevated (Borzym-Kluczyk et al., 2008). In salivary gland tumour tissue, the activity of HEX, HEX-A, HEX-B, GAL and FUC was considerably higher compared to healthy tissue. The activity of all examined lysosomal exoglycosidases (HEX, HEX-A, HEX-B, GLU, GAL, MAN and FUC) in the serum of patients with salivary gland tumours was significantly higher compared to the serum of healthy controls. The saliva of patients with salivary gland tumours also showed significantly higher activity of HEX, HEX-A, GLU, GAL, MAN, and FUC, and a downward trend in HEX-B activity compared to that of healthy controls. The assessment of serum and saliva HEX levels in patients with salivary gland tumours could be potentially employed in diagnosing and monitoring salivary gland tumours (Bierc et al., 2010).

#### 4.6.8 Laryngeal cancer

Laryngeal cancer is one of the most common tumours of the head and neck (Olszewska et al., 2012). Both surgical treatment and radiotherapy are unsatisfactory (Olszewska et al., 2012). Olszewska et al. (2012) attempted to evaluate MAN and GAL activity in squamous cell carcinoma of the larynx. The authors observed higher MAN activity in all laryngeal cancer samples compared to healthy tissue samples. The mean MAN activity was 3.702 ± 1.3245 nkat/g wet tissue compared to the control group (1.614 ± 0.8220 nkat/g wet tissue). The authors also showed significantly higher GAL activity in laryngeal cancer compared to healthy tissue samples. The mean GAL activity was 3.383 ± 2.1980 nkat/g wet tissue compared to the control group (2.137 ± 1.3685 nkat/g wet tissue).

### 4.6.9 Other tumours

Severini et al. determined HEX and GLU activity in the serum of patients with breast and gastrointestinal cancer and in healthy individuals (Severini et al., 1995). HEX levels elevated above the normal range (45.6 U/L) were observed in 78% patients with breast cancer, 100% with gastric cancer, 70% with colorectal cancer, 100% with liver cancer, and 100% with pancreatic cancer. In patients with breast and gastric cancer, HEX showed good specificity (90% and 91%, respectively) and sufficient sensitivity (78% and 100%, respectively) as a potential tumour marker. GLU proved less sensitive and was significantly elevated (in all patients) only in the case of pancreatic cancer (Severini et al., 1995).

### 4.7 Nasopharyngeal disease

Diagnosis of chronic inflammatory diseases of the upper respiratory tract, including chronic tonsillitis and nasal polyps, is primarily based on clinical examination, endoscopic evaluation, imaging studies, and histopathological assessment. However, currently available diagnostic approaches provide limited information regarding ongoing extracellular matrix remodeling and tissue degradation. Therefore, biomarkers reflecting local glycoconjugate turnover may provide useful complementary information regarding disease activity and tissue remodeling (Fokkens et al., 2020; Windfuhr et al., 2016).

#### 4.7.1 Nasal polyps

Nasal polyps are benign protrusions of the swollen mucous membrane from the nasal passages towards the nasal cavity, which can occur in various stages, from a single lesion to a diffuse one (Preś et al., 2004). The formation of polyps is very often related to chronic (lasting more than 12 weeks) inflammation of the nasal and sinus mucosa. There are various theories regarding the pathogenesis of nasal polyps; however, none have yet been fully confirmed (Chojnowska, 2009; Morimoto et al., 1971). One of these is impaired catabolism of glycoconjugates in the nasal mucosa and sinuses.

Chojnowska et al. observed significantly lower GAL and FUC activity, as well as a trend towards reduced MAN and GLU expression, in nasal polyp tissue compared to healthy mucosa. However, there were no differences in specific GAL, MAN and FUC activity in nasal polyp tissue compared to control mucosa (Chojnowska et al., 2013). The authors explain this by deregulation of inflammatory mechanisms. During a fully symptomatic inflammation, such as tonsillitis (Chojnowska et al., 2013; Steinke et al., 2004), an increase in tissue remodelling was observed, expressed as increased catabolism of glycoconjugates, which manifested as higher activity of lysosomal exoglycosidases. The decrease in the activity of exoglycosidases involved in the catabolism of oligosaccharide chains of glycoconjugates in nasal polyp tissue is an argument against classifying nasal polyps as a fully inflammatory disease (Bernstein, 2001; Al-Bazzaz et al., 1981; Bernstein, 2005).

#### 4.7.2 Chronic tonsillitis

Chronic tonsillitis presents as a sore throat lasting more than 3 months, accompanied by symptoms of inflammation and enlarged lymph nodes. The cause is a mixed infection with both aerobic and anaerobic bacteria, which can survive in the throat as a biofilm. Hypertrophy and tonsillitis are indications for tonsillectomy (Brodsky and Poje, 2014; Paulussen et al., 2000), but an increasing number of clinicians believe that the indications for their removal should be limited (Brodsky and Poje, 2014; Paulussen et al., 2000; Zhang et al., 2003). Popko et al. examined the activity and specific activity of HEX, HEX-A, HEX-B, GLU, GAL, MAN and FUC in both hypertrophied and inflamed palatine tonsil tissue (in children and adults) (Popko, 2007). The activity and specific activity of each of the examined exoglycosidases were significantly higher in each of the three pathological tissue groups compared to healthy tonsil tissue. No significant differences in the inflamed tonsils were found based on age. This suggests that complete removal of the palatine tonsils (tonsillectomy) is justified in cases of both hypertrophy and inflammation (in childhood) (Popko, 2007).

### 4.8 Musculoskeletal diseases

Joint diseases cause serious medical problems in several million people worldwide (Parnetti et al., 2014). Osteoarthritis (OA) is the most common and increasingly prevalent joint disease (Dieppe, 2000). Diagnosis and monitoring of rheumatic diseases frequently rely on clinical assessment, imaging studies, inflammatory markers, and disease-specific autoantibodies (Smolen et al., 2016). However, these measures may not adequately reflect ongoing extracellular matrix degradation within affected tissues (Mobasheri et al., 2017). Biomarkers associated with glycoconjugate turnover could therefore provide complementary information regarding disease activity. Rheumatic diseases are characterized by chronic inflammation and progressive degradation of connective tissue components, including collagen, proteoglycans, and glycosaminoglycans, which contribute to tissue destruction and functional impairment. These pathological processes are accompanied by profound alterations in extracellular matrix turnover and remodeling (Goldring and Goldring, 2007; Denton and Khanna, 2017).

#### 4.8.1 Inflammatory joint diseases

In inflammatory joint diseases (such as osteoarthritis, rheumatoid arthritis, Lyme arthritis and juvenile idiopathic arthritis), the destruction of joint cartilage is a multifactorial process that also involves disorders of glycoconjugate catabolism (Popko et al., 2012). In the course of arthritis, the activity of lysosomal exoglycosidases increases, releasing individual monosaccharides from the non-reducing ends of oligosaccharide chains in synovial tissue, articular cartilage and synovial fluid (Popko et al., 2012). Popko et al. found that an elevation of HEX activity in synovial fluid to more than 10 μkat/kg protein suggests rheumatoid arthritis (Popko et al., 2012). Pancewicz et al. showed that HEX, GAL and MAN activity were significantly higher in the serum of patients with Lyme arthritis compared to healthy individuals and normalised after treatment with doxycycline (Pancewicz et al., 2006). HEX activity in the serum of patients with Lyme arthritis was significantly higher compared to controls (p < 0:001). After 4 weeks of treatment with doxycycline, this activity tended to decrease, but was still higher than in healthy individuals. GAL and MAN activity in patients with Lyme arthritis before therapy was elevated compared to the control group; after treatment, it fell to almost the same level as that observed in healthy individuals. These changes were not statistically significant, but the trend was consistent. It is suggested that HEX may be a sensitive enzymatic marker for monitoring disease progression and treatment efficacy in Lyme arthritis (Pancewicz et al., 2006).

Another study by Pancewicz et al. compared the activity of HEX, GLU, GAL, MAN and FUC in the serum and synovial fluid of patients with Lyme disease, rheumatoid arthritis and juvenile idiopathic arthritis. A significant increase in the activity of all exoglycosidases in serum and synovial fluid was observed in patients with various forms of arthritis compared to controls. The ratio of exoglycosidase activity in synovial fluid to exoglycosidase activity in serum was above two in patients with Lyme disease, but not in patients with rheumatoid arthritis or juvenile idiopathic arthritis. Since the synovial membrane is the primary source of exoglycosidases in the joint, this finding supports the therapeutic value of synovectomy in chronic Lyme arthritis with knee effusion. Serum HEX activity assessment can be used to monitor the course of Lyme arthritis and the efficacy of treatment (Pancewicz et al., 2009).

#### 4.8.2 Systemic sclerosis

Systemic sclerosis is a systemic connective tissue disorder characterised by progressive fibrosis of the skin and internal organs, salivary hypofunction, abnormalities in the morphology and function of blood vessels, and immune system disorders. Its aetiology is not well understood (Volkmann et al., 2023). Epidemiological studies indicate that women are 3–4 times more likely to develop systemic sclerosis, with the peak incidence occurring between the ages of 30 and 50 (Efrimescu et al., 2023). Knaś et al. found significantly higher HEX, HEX-A, HEX-B and GLU activity in the unstimulated saliva of patients with systemic sclerosis compared to the control group (Knaś et al., 2014). A negative correlation was also demonstrated between HEX-B activity and unstimulated saliva secretion per minute, suggesting that disorders of glycoconjugate catabolism contribute to salivary hypofunction in patients with systemic sclerosis. GLU is secreted mainly by inflammatory cells (neutrophils, macrophages), which means that increased enzyme activity in saliva may be an indicator of early, clinically asymptomatic damage to the parotid glands in the course of systemic sclerosis (Knaś et al., 2014).

## 5 The effect of drugs on lysosomal exoglycosidase activity

Pharmacological treatment may influence lysosomal enzyme activity through modulation of inflammation, oxidative stress, cellular turnover, and tissue remodeling. However, current evidence regarding the effects of antihypertensive agents, antidiabetic drugs, immunosuppressants, or anticancer therapies on exoglycosidase activity remains limited. Future studies should investigate whether changes in enzyme activity can serve as surrogate markers of treatment response (Frangogiannis, 2015).

## 6 Advantages and disadvantages of exoglycosidases as biomarkers

A common feature of many diseases discussed in this review is disruption of glycoconjugate and glycosaminoglycan homeostasis. Glycosaminoglycans are essential components of extracellular matrix, basement membranes, and cell surfaces. Chronic inflammation, oxidative stress, ischemia, fibrosis, and malignant transformation may promote extracellular matrix remodeling and accelerate glycoconjugate degradation (Theocharis et al., 2016). Increased activity of lysosomal exoglycosidases may therefore represent a universal biochemical signature of pathological tissue remodeling rather than a disease-specific phenomenon. This concept provides a unifying mechanistic explanation for the observed enzyme alterations across diverse disease entities.

Lysosomal exoglycosidases are typical indicator enzymes, i.e., compounds that occur intracellularly and whose concentration (activity) in body fluids increases as a result of cell damage. Exoglycosidases belong to organ-nonspecific enzymes—they have been identified in the liver, kidneys, gastric mucosa, spleen, brain, placenta, lungs, and skin fibroblasts (Zwierz et al., 1992). As a result of cell damage, they enter not only the bloodstream but also urine, saliva, joint fluid, and cerebrospinal fluid, enabling their clinical use. The presence of lysosomal exoglycosidases in urine and saliva warrants special attention, as these are biological fluids collected non-invasively. Exoglycosidases are not denatured at the acidic pH of urine, and their secretion increases significantly in kidney diseases (Zalewska-Szajda et al., 2019b). They are also relatively stable in saliva, allowing them to be used for both oral and systemic diseases (Knaś et al., 2007). Non-invasive sampling reduces patient anxiety during medical examinations, particularly in young children and the elderly. Saliva and urine can also be used in patients with blood-clotting disorders. Another unquestionable advantage of lysosomal exoglycosidases is the simplicity and low cost of assessing their activity in biological samples. The colorimetric method based on p-nitrophenol measurement can be used in routine analytical laboratories. Although exoglycosidases are highly sensitive, they are also relatively non-specific. They may indicate several diseases, including liver disease, AUD, nicotine addiction, kidney disease, cardiovascular disease, diabetes, cancer, nasopharyngeal disease, and musculoskeletal disorders (Knaś et al., 2007; Maciejczyk et al., 2017; Waszkiewicz et al., 2014). However, lysosomal exoglycosidases may have a particular application in addiction medicine. Compared to routine biomarkers, they are more sensitive and specific to alcohol-induced damage. Importantly, lysosomal exoglycosidases (especially HEX and its isoenzymes) show a greater correlation with acute ethanol exposure and are less dependent on individual factors (age, gender, comorbidities).

Further studies are needed to evaluate the diagnostic utility of lysosomal exoglycosidases in larger populations with different clinical conditions. Although reference values for selected lysosomal exoglycosidases have been established in healthy adults and children, available studies remain limited with respect to population size, age range, biological fluids examined, and analytical methodology (Zalewska-Szajda et al., 2018b; Müller et al., 2010; Mordaunt et al., 2024). Age-dependent reference intervals have been established for urinary HEX, HEX A, HEX B, FUC, GAL, GLU, and MAN in healthy children, demonstrating significantly higher enzyme activities during early childhood and emphasizing the need for age-specific reference ranges (Zalewska-Szajda et al., 2018b; Zalewska-Szajda et al., 2019c). Similarly, Oláh et al. demonstrated that urinary HEX activity is highest during the first years of life and gradually decreases throughout childhood, supporting the importance of age-specific reference values (Oláh et al., 1994). In healthy adults, urinary HEX reference intervals have been proposed according to age and sex, whereas recent large-scale studies have established reference intervals for several lysosomal exoglycosidases in leukocytes and plasma using standardized statistical approaches (Mordaunt et al., 2024; Liu et al., 2021)., An overview of the currently available published reference values and reference intervals is presented in Table 2. Despite these advances, comprehensive harmonized reference intervals applicable across different biological fluids and analytical platforms remain unavailable. Future multicenter studies are therefore required to establish standardized reference intervals accounting for biological variability related to age, sex, and methodological differences.

**TABLE 2:** Published reference values and reference intervals for lysosomal exoglycosidases in healthy populations. Reference values are presented in the original units reported by the respective studies. Direct comparison between studies is limited because of differences in analytical methods, assay substrates, normalization procedures, study populations, and statistical approaches used to establish reference intervals.

Enzyme	Population	Age (years)	References value	Unit	References
Blood					
Dried bood spots					
GAL	Healthy individuals		4.04–29.65	μmol/L blood/h	(Müller et al., 2010)
Blood - Leukocytes					
HEX	Healthy individuals	Children and adults (all ages)	15.0–49.0	nmol/min/mg protein	(Mordaunt et al., 2024)
HEX A			4.0–15.0		
FUC			0.58–2.70		
GAL			7.20–32.00		
GLU			0.53–3.10		
MAN			4.30–23.00		
Blood - plasma					
GLU	Healthy individuals	Children and adults (all ages)	0.08–1.10	nmol/min/mL	(Mordaunt et al., 2024)
MAN			0.20–1.40		
Urine					
HEX	Adults, males	20–59	<19.4	U/L	(Liu et al., 2021)
		60–79	<22.3		
	Adults, females	20–59	<15.7		
		60–79	<21.4		
HEX	Healthy children (0.5–17.9 years)	Age-specific	0.028–1.081	pKat/µg creatinine	(Zalewska-Szajda et al., 2018b; Zalewska-Szajda et al., 2019c)
HEX A			0.005–0.496		
HEX B			0.021–0.684		
FUC			0.019–1.205		
GAL			0.010–1.372		
GLU			0.008–1.420		
MAN			0.021–1.949		

## 7 Conclusion

Unlike previous reviews that focused mainly on individual enzymes or selected disease entities, the present article integrates evidence from hepatology, nephrology, cardiology, oncology, addiction medicine, and rheumatology. Particular attention is given to recently published studies concerning diet-induced liver injury, systemic sclerosis, salivary gland tumors, and pediatric nephrology. The review also discusses emerging concepts such as isoenzyme profiling and fluid-specific biomarker applications.

Lysosomal exoglycosidases are enzymes whose activity in body fluids is being investigated as a potential biomarker for many systemic diseases. The advantage of these hydrolases is their widespread presence and stability in various tissues and body fluids, as well as the ease of non-invasive detection in serum, urine or saliva. Many studies indicated changes in exoglycosidase activity in the course of various diseases, including liver and kidney disease, diabetes, hypertension, malignant tumours, and in people who chronically alcohol use disorder and smoke tobacco. For example, in acute hepatitis and alcoholic liver disease, there is a significant increase in the activity of these enzymes, and α-L-fucosidase was shown to be a sensitive marker of hepatocellular carcinoma. Nephropathies are characterised by an early increase in urinary excretion of exoglycosidases, reflecting damage to the renal tubules, although their levels vary depending on age, sex and exercise. In diabetes (types 1 and 2), elevated HEX and GLU activity is found in saliva, serum, and urine, especially in cases of complications. The ratio of HEX-A/HEX-B activity reflects the severity of the disease. In hypertension, increased urinary excretion of HEX correlates with blood pressure, suggesting early nephropathic changes. Elevated exoglycosidase activity is observed in many tumours; some (e.g., HEX-A, FUC) show high diagnostic sensitivity with moderate specificity. Chronic AUD results in a significant increase in HEX activity (especially HEX-B) in serum, urine and saliva, with sensitivity and specificity exceeding traditional indicators of liver damage; these indicators normalise after several days of abstinence, enabling sobriety to be monitored. Smoking also increases urinary excretion of HEX, indicating subclinical renal damage.

One of the main obstacles hindering the clinical implementation of lysosomal exoglycosidases is the lack of standardized reference intervals. Available studies show that enzyme activity can be influenced by age, gender, physical activity, and in some cases, blood pressure, metabolic status, and selected environmental factors. The lack of universally accepted reference intervals for serum, urine, and saliva is a gap we consider one of the main barriers to clinical application. Unfortunately, currently available studies are highly heterogeneous and do not provide sufficient data to reliably establish reference ranges for all age groups and biological fluids. Establishing age- and fluid-specific reference values should be a priority for future research.

Given their relatively low disease specificity, lysosomal exoglycosidases are unlikely to replace existing biomarkers when used individually. Greater diagnostic value may be achieved through combined assessment of multiple exoglycosidases, their isoenzymes, and established clinical biomarkers. Future studies should evaluate multivariate diagnostic models incorporating exoglycosidases together with inflammatory markers, imaging findings, and clinical variables. Machine-learning approaches may further improve disease classification and prediction accuracy.

Despite promising results, the use of exoglycosidases as biomarkers requires further research. To date, most studies suffer from numerous limitations (heterogeneous populations, comorbidities), and thus standardisation and validation in larger populations are necessary to confirm the true diagnostic value of these indicators. Future studies should verify the usefulness of exoglycosidases in screening (early detection of cancer and nephropathy), disease monitoring and personalised therapy.
