# Supplementary material for: Lysosomal exoglycosidases as biomarkers of systemic diseases – what’s new?
Source: Front Mol Biosci. 2026 Oct 16;13:1891977. doi: 10.3389/fmolb.2026.1891977 (PMC13720770; doi:10.3389/fmolb.2026.1891977)
Supplement: Supplementary file 1 [file Table1.docx]

Table S1. Detailed description of the clinical significance of lysosomal exoglycosidases.

| Disease | Results | Conclusions |
| --- | --- | --- |
| Liver diseases | | |
| Acute hepatitis | Hultberg et al. (58) demonstrated that serum HEX activity was significantly elevated in acute hepatitis A and slowly decreased with treatment. Positive correlations were found between serum HEX activity and ALT activity (r = 0.44, p < 0.01) and AST activity (r = 0.40, p < 0.02). The changes in HEX activity in acute viral hepatitis B were similar. Positive correlations were found between serum HEX activity and ALT activity (r = 0.33, p < 0.05), AST activity (r = 0.46, p < 0.005) and alkaline phosphatase activity (ALP; r = 0.4, p < 0.02), as well as bilirubin concentration (r = 0.61, p < 0.001). | Elevated HEX is interpreted as the result of impaired function of the liver’s reticuloendothelial cells (Kupffer cells). β-hexosaminidase may be an indicator of the function of the reticuloendothelial system in the course of viral hepatitis, but it does not replace classic liver tests. |
| Chronic hepatitis | Ohta et al. (61) indicate that serum GLU activity was significantly higher in patients with liver cancer, cirrhosis and chronic active hepatitis compared to the control group. A positive correlation was found between GLU activity and the degree of liver cell necrosis determined by histological observation. It was confirmed that necrotic liver cells are the source of GLU in serum. Eight out of ten patients with labile and elevated serum GLU activity showed progression of chronic hepatitis. | Studies indicated that GLU activity measurement can be applied to assess current liver cell necrosis and predict the histological progression of chronic hepatitis. |
| Alcoholic liver disease | Faber et al. (63) assessed the activity of lysosomal exoglycosidases in the serum of patients with alcoholic liver disease confirmed by biopsy or a history of chronic AUD. The authors reported a significant increase in the activity of HEX (1.4-fold), GLU (2.1-fold), acid MAN (2.2-fold), intermediate MAN (1.6-fold) and FUC (2.3-fold) in the serum of patients with liver disease compared to the control group. MAN activity was significantly higher (p < 0.001) when measured in an acidic environment compared to measurements at neutral pH.  Reglero et al. (64) indicated significantly higher α-L-fucosidase and β-N-acetylglucosaminidase activity in serum in patients with diabetes, cirrhosis and gastric cancer compared to the control group, and no significant differences in the activity of other glycosidases.  Marshall et al. (65) demonstrated that serum from patients with alcoholic cirrhosis and liver metastases may contain elevated levels of desialylated glycoproteins, thus providing evidence of abnormalities in the carbohydrate recognition system. In patients with mechanical jaundice, the levels of these glycoproteins were similar to those of healthy individuals. | The authors suggest that elevated enzyme levels may stem from reduced clearance of glycoproteins from the circulation, which is typical of liver disease, and not necessarily from increased enzyme production. Measuring serum MAN activity may be an additional indicator for monitoring alcohol-related liver disease, but it does not replace standard liver tests.  The authors suggest that cirrhosis alters lysosome integrity and membrane permeability. Elevated levels of α-L-fucosidase and β-N-acetylglucosaminidase in serum may suggest their potential role as biochemical markers in the diagnosis of patients with diabetes, cirrhosis and gastric cancer.  The authors concluded that increased levels of desialylated glycoproteins are associated with the extent of liver damage, indicating their potential use as a biochemical marker of hepatocyte damage, especially in cirrhosis and liver metastases. |
| Diet-induced liver damage | Żebrowska-Gamdzyk et al. (54) demonstrated that a high-protein diet based on whey proteins may impair liver function. The authors investigated the effect of whey protein concentrate (WPC-80) on glycoconjugate catabolism, as well as selected markers of oxidative stress and liver inflammation. In liver homogenates, the authors examined the activity of HEX, GLU, GAL, MAN, FUC, as well as the levels of reduced glutathione (GSH), malondialdehyde (MDA), interleukin-1β (IL-1β) and transforming growth factor-β1 (TGF-β1). Significantly higher HEX, GLU, MAN and FUC activity was found in the livers of rats receiving WPC-80 compared to the controls. Serum alanine aminotransferase (ALT) and aspartate aminotransferase (AST) concentrations were significantly higher in animals supplemented with WPC-80 at 0.5 g/kg body weight for 21 days. Increased levels of GSH, MDA, IL-1β and TGF-β1 were also observed in the same group of animals. WPC-80 contributes to intense liver tissue remodelling and induces oxidative stress, particularly at a dose of 0.5 g/kg body weight. | Long-term administration of WPC-80 to male rats resulted in intensified glycoconjugate catabolism and induced oxidative stress and liver inflammation. These results suggest that excessive consumption of WPC-80 may impact liver metabolism, which may be relevant in the context of long-term use of protein supplements. |
| Alcohol use disorder and substance-related disorders | | |
| Alcohol use disorder | Waszkiewicz et al. (78) observed that even a single high dose of ethanol can increase the activity of total β-HEX, β-HEX A, and β-HEX B in serum, as well as total β-HEX in saliva and β-HEX A in urine. Acute intake (6 hours) of a high but tolerable dose of alcohol (120–160 g) significantly increases the activity of total HEX and the HEX-A and HEX-B isoenzymes in serum, as well as total HEX in saliva and HEX-A in urine in infrequent drinkers. It was shown that the increase in total HEX activity in serum and urine is mainly caused by the secretory isoenzyme HEX-A.    Waszkiewicz et al. (79) studied exoglycosidase activity on days 1, 15, 30 and 50 of abstinence following chronic alcohol intake.  In alcohol-dependent groups, a 2–3-fold increase in MAN activity, a 2–6-fold increase in FUC, an 8–25-fold increase in HEX-A, and a 19–40-fold increase in GLU were found compared to the control group. The highest secretion of GLU and HEX-A was observed on day 1 of abstinence, while on day 50, the highest secretion was observed for GLU, FUC and MAN. GLU and HEX-A activity correlated positively with the number of days since last alcohol intake. The activity and secretion of FUC and MAN correlated positively with the duration of AUD.  Hultberg et al. (80) analysed HEX isoenzymes in the serum of people with alcohol use disorder (AUD) hospitalised for detoxification compared to abstinent people with AUD and a reference group of non-drinkers. Serum HEX-B was significantly elevated in all patients hospitalised for detoxification. In the abstainers group, only one had elevated levels of HEX-A, HEX-B, and total HEX compared to the control group. HEX-B had a half-life of 2 to 4 days in hospitalised patients, while the half-lives of HEX-A and gamma-glutamyltransferase (GT) differed significantly. No clear pattern of decrease in HEX-A and GT was found, as in the case of HEX-B. HEX-B correlated significantly with total HEX and HEX-A, while HEX-A correlated with HEX, AST and GT (r = 0.99, p < 0.001).  Waszkiewicz et al. (81) examined HEX-A% (percentage of β-HEX A isoenzyme to total β-HEX) and HEX-B% (percentage of β-HEX B isoenzyme to total β-HEX) in the saliva of people with AUD with periodontal disease. HEX-A% was significantly higher and HEX-B% lower in the alcohol-dependent smoker group compared to the healthy group. The high AUC value for 𝛽-HEX A% (0.937) in the AS group [alcohol-dependent smokers], excellent sensitivity (96%) and specificity (91%) of 𝛽-HEX A% and its correlation with the gingival index (GI) indicate the high value of 𝛽-HEX A% as an excellent marker of periodontal disease.  Ptaszyńska-Sarosiek et al. (83) assessed the activity of HEX, HEX-A and HEX-B in blood, urine, cerebrospinal fluid and vitreous humour in alcohol poisoning fatalities (alcohol concentration of 4‰ and above) compared to sudden death cases. The highest serum HEX activity was found in both the study and control groups. Significantly lower HEX, HEX-A and HEX-B activity was observed in the urine of alcohol poisoning victims compared to those who died sober. HEX and its isoenzymes A and B were higher only in blood serum in both study groups, and this may be related to liver damage. In ethanol poisoning fatalities, lower HEX, HEX-A, and HEX-B activity was observed in urine, cerebrospinal fluid, and vitreous humour compared to blood serum, which may be evidence of blood–organ barrier damage. Decreased HEX activity in urine can be explained by harmful alcohol use rather than AUD. In alcohol poisoning fatalities, significantly lower levels of HEX, HEX-A and HEX-B in urine than in blood serum may indicate significant kidney damage caused by ethanol and its metabolites, particularly acetaldehyde.  Taracha et al. (84) investigated the usefulness of measuring HEX activity in the urine of patients undergoing methadone substitution treatment to identify cases of AUD. It was found that methadone, illicit drugs and HIV seropositivity did not affect the activity of this enzyme. β-HEX activity was significantly higher in patients who admitted to occasional heavy drinking compared to those who declared abstinence (p = 0.014) and compared to healthy controls (p = 5 × 10⁻⁸). β-HEX activity was still lower than in detoxified alcohol-dependent subjects (p = 0.024). In the group of opioid-dependent individuals participating in the methadone substitution programme, the sensitivity of the test was 87% and the specificity was 80%. | HEX activity varied depending on the type of biological sample, suggesting different dynamics of alcohol metabolism in the body. The variability of HEX activity in response to alcohol intake may suggest its potential use as a biomarker in monitoring alcohol intake.  GLU activity is the most useful marker of chronic alcohol use disorder. FUC and MAN activity may serve as an indicator of AUD. HEX-A activity is associated with chronic alcohol intake.  .  The results suggest that salivary exoglycosidases may be useful in the diagnosis of chronic alcohol intake and use disorder.  HEX-B was found to be a useful marker of AUD.  Studies indicated that increased levels of 𝛽-HEX A% in saliva may be an excellent marker of periodontal disease.  Studies revealed the highest HEX-A activity in the urine of fatal ethanol poisoning victims compared to HEX and HEX-B, which may be explained by ethanol-induced inflammatory kidney damage. The decrease in HEX activity and its isoenzymes in urine can also be explained by lower vasopressin (antidiuretic hormone) secretion in people who consume large amounts of ethanol.  Measuring β-hexosaminidase activity in urine may help diagnose AUD in opioid-dependent individuals participating in methadone substitution programmes. This test shows high sensitivity and specificity, making it a promising tool for monitoring AUD in this patient population.  It was found that methadone, illicit drugs and HIV seropositivity did not affect the activity of this enzyme. However, HEX activity was significantly higher in patients who admitted to occasional heavy drinking compared to patients who declared abstinence and healthy individuals. Due to the test’s sensitivity, measuring HEX activity in urine can help assess the effects of AUD treatment. |
| Nicotine addiction | Hultberg et al. (90) found that urinary HEX excretion in 203 apparently healthy men and women correlated significantly with tobacco consumption (r = 0.26, p < 0.001), whereas urinary creatinine excretion did not differ between smokers and non-smokers.  Kawada et al. (92) studied workers exposed to inhalation of pigment powder containing cadmium.  Cadmium concentration in urine (Cd) in April and September ranged from 0.2 to 9.5 μg/g creatinine in April (geometric mean approx. 0.7 µg/g cr) and 0.5 to 7.0 μg/g cr in September (geometric mean approx. 1.2 µg/g cr).  Correlation between Cd concentration and N-acetyl-β-D-glucosaminidase (NAG) activity  in April: r = 0.261 (n = 61), in September: r = 0.389 (n = 50). Correlation between Cd concentration and β-2-microglobulin (BMG)  in April: r = 0.241 (n = 63), in September: r = 0.115 (n = 50). | Individuals who smoked more than 10 g of tobacco per day excreted significantly more HEX in urine than non-smokers, which may indicate the presence of low-grade kidney damage in smokers.  NAG activity in urine correlates better with cadmium concentration than β-2-microglobulin, even at relatively low cadmium concentrations (< 10 μg/g creatinine). NAG may be a more sensitive indicator of cadmium exposure in the workplace than BMG. One reason for the increase in exoglycosidase activity in the urine of smokers may be smoking-related cadmium exposure. |
| Kidney diseases | | |
| Ureteropelvic junction obstruction | Taranta-Janusz K. et al. (109) found that the activity of lysosomal exoglycosidases in urine was significantly higher in children with ureteropelvic junction obstruction than in healthy children. The activity of most urinary exoglucosidases was strongly positively correlated with the urine albumin/creatinine ratio. This means that the higher the enzyme activity, the higher the level of albumin in the urine, suggesting damage to the renal tubules. | Urine exoglycosidases may be potential markers of tubulointerstitial renal injury in children with ureteropelvic junction obstruction. They can assist in assessing the extent of damage and making informed clinical decisions (for instance, determining whether surgery is necessary). |
| Tubular injury in patients with a solitary functioning kidney | Taranta-Janusz et al. (110) demonstrated that the activity of HEX, HEX-A, HEX-B, FUC, GAL, MAN, and GLU in urine was significantly higher in children with tubular injury and a solitary functioning kidney than in healthy patients (p < 0.05). Compared to the control group, patients with congenital solitary functioning kidney (cSFK) showed significantly higher activity levels for all exoglycosidases (p < 0.05). Similar results were observed in children with acquired solitary functioning kidney (aSFK), excluding HEX-A (p > 0.05). Very strong positive correlations were found between the activity of all urinary exoglycosidases and the albumin/creatinine ratio (p < 0.001). The median estimated glomerular filtration rate (eGFR) correlated negatively with age in patients with SFK (r = –0.33, p < 0.01). A negative correlation was demonstrated between HEX, its isoenzymes HEX-A and HEX-B (pKat/mL), and eGFR (r = –0.29, r = –0.25, r = –0.29, p < 0.05, respectively) in children with SFK. | These findings may indicate damage to the renal tubules in children with a solitary functioning kidney, with high (Group I) and low (Group II) glomerular filtration rates.  Lysosomal enzymes may be useful biomarkers of tubular injury in children with a solitary functioning kidney. |
| Hydronephrosis | Taranta-Janusz et al. (109) demonstrated that the activity of HEX, HEX-A, HEX-B, FUC, GAL, MAN and GLU in urine was significantly higher in children with hydronephrosis caused by ureteropelvic junction obstruction than in healthy children. This means that even children without obvious symptoms of kidney failure develop tubular injury. The highest enzyme levels were recorded in children with more severe hydronephrosis, who were eligible for surgical treatment. This suggests that exoglycosidase activity may be useful in differentiating the severity of hydronephrosis. The activity of exoglucosidases was correlated with urinary albumin excretion, supporting their significance as markers of tubular injury in patients with hydronephrosis. | These enzymes (e.g. α-fucosidase, β-hexosaminidase) may serve as sensitive and early markers of tubulointerstitial injury, even before changes in standard renal parameters (e.g. creatinine) become apparent. The authors emphasise that testing for exoglucosidase activity in urine can help monitor children with hydronephrosis and enable early diagnosis of cases requiring surgical intervention. |
| Renal tumours | Liubimova et al. (119) investigated the usefulness of assessing exoglycosidase activity in urine to predict nephrotoxicity in children with malignant solid tumours who received polychemotherapy with cisplatin at 100 mg/m^2^ or ifosfamide at 9 g/m^2^. After chemotherapy, hyperenzymuria persisted, with significantly higher levels of HEX, GTP and alanine aminopeptidases (ANAases) in the urine compared to baseline levels. Treatment with fosfamide significantly increased HEX activity in urine compared to patients treated with cisplatin. | The authors concluded that HEX is a more sensitive marker of renal tubular injury caused by cytostatic drugs than urea and the albumin/creatinine ratio. |
| Kidney transplantation | Hultberg et al. (121) studied HEX concentration and HEX-B percentage using the enzyme immunoassay (EIA) method in the urine of kidney transplant patients treated with cyclosporine A, prednisolone and azathioprine. In patients after a kidney transplant without complications, a significant decrease (approaching a normal level) in HEX activity in urine and no change in the percentage of HEX-B were observed. | According to the authors, increased HEX activity in urine is an early indicator of kidney transplant rejection. |
| Diabetic nephropathy | Romero et al. (122) reported that HEX activity in the urine of children in the early stages of diabetic nephropathy is twice as high as HEX activity in the urine of healthy children. In the group treated with captopril, microalbuminuria decreased after 6 months. In contrast, it did not change significantly in the control group (p = 0.044 for the change in the treatment group, p = 0.027 for the change between groups). The filtration fraction in the treatment group decreased from approximately 0.23±0.03 to 0.22±0.04, while it increased in the control group from 0.22±0.04 to 0.25±0.04 (the percentage change between the groups was significant, p=0.032). | In normotensive patients with type 2 diabetes, the presence of microalbuminuria indicates an early stage of diabetic nephropathy. Treatment with an angiotensin-converting enzyme (ACE) inhibitor resulted in a significant reduction in microalbuminuria and a decrease in the filtration fraction, suggesting a beneficial effect on glomerular haemodynamics and renal protection. Although the patients included in the study were normotensive, a reduction in diastolic blood pressure during treatment was associated with an improvement in renal parameters. This means that the mechanism of kidney protection by ACE inhibitors applies not only to patients with hypertension but also to those without it. The use of ACE inhibitors in the early stages of diabetic nephropathy (at the microalbuminuria stage) may delay progression to overt nephropathy and chronic renal failure.  This study provided early evidence that ACE inhibitor therapy in normotensive patients with type 2 diabetes and microalbuminuria may protect the kidneys from developing diabetic nephropathy. |
| Cardiovascular diseases | | |
| Hypertension | Zalewska-Szajda et al. (127) reported that HEX-A activity in the urine of children with prehypertension (when systolic blood pressure is between 120 and 139 mm Hg and/or diastolic blood pressure is between 80 and 89 mm Hg) was significantly higher than in children with normal blood pressure. Furthermore, the group of children with prehypertension showed a positive correlation between HEX and HE-A and systolic blood pressure (r = 0.17, p < 0.05; r = 0.21, p < 0.05, respectively). The AUC differentiating these two groups was 0.616 for HEX-A (cut-off value of 29.351 pKat/mL; sensitivity 51.2%, specificity 71.8%) and 0.589, with a cut-off value of 0.054 pKat/μgCr (sensitivity 31.7%, specificity 86.3%) .  In turn, Zalewska-Szajda et al. (101) demonstrated that the activity of HEX and its isoenzymes (expressed as pKat/μg creatinine) was statistically significantly higher in the youngest children (0.5–2.9 years) than in adolescents (15.0–17.9 years).  A negative correlation between age and the activity of the enzymes was found: HEX: r = –0.24, p < 0.001, HEX-A: r = –0.20, p < 0.01, HEX-B: r = –0.26, p < 0.001.  Similarly, FUC, GAL, GLU, and MAN activity was significantly higher in the youngest children compared to adolescents (p<0.001). A negative correlation between age and the activity of the enzymes was also demonstrated: FUC: r = –0.36, p < 0.0001, GAL: r = –0.36, p < 0.0001, GLU: r = -0.35, p < 0.0001, MAN: r = –0.35, p < 0.0001. Enzyme activity was independent of sex in all age groups.    Alderman et al. (139) investigated the level of NAG excretion in urine in patients with hypertension and the effect of antihypertensive treatment on these levels. Before treatment, the mean urinary NAG level in hypertensive patients was 65 ± 61 nmol/g creatinine per hour, whereas it was 29 ± 16 nmol/g creatinine per hour in the control group (p < 0.01). After three months of treatment, NAG levels did not change significantly, remaining at 54 ± 54 nmol/g creatinine per hour. After 12 months of treatment, NAG level decreased to 45 ± 28 nmol/g creatinine per hour (p < 0.01 compared to baseline). The level of NAG in urine correlated directly with systolic blood pressure (p < 0.01), but not with diastolic blood pressure, age, sex or race. | HEX-A is one of the examined enzymes that may serve as a potential marker to identify children with high blood pressure among their healthy peers.  The activity of lysosomal exoglycosidases in urine in children and adolescents decreases with age when its activity is corrected by creatinine. These enzymes are secreted in minimal amounts in the urine of healthy children, making age-specific reference values useful in differentiating between healthy and pathological kidneys. As traditional indicators of kidney function (e.g. creatinine, GFR, creatinine clearance) may fail to detect early changes, exoglycosidase activity may be a promising marker of early renal tubular injury. The reference values established in the study may serve as a basis for later studies of renal disease in children and adolescents.  Patients with hypertension showed significantly higher levels of NAG in urine, suggesting the presence of subclinical kidney injury. Decrease in NAG levels after treatment: Effective antihypertensive treatment resulted in a decrease in urinary NAG levels, suggesting improved kidney function. Measuring urinary NAG levels may be a valuable marker for assessing kidney injury in patients with hypertension. |
| Atherosclerosis | Hultberg et al. (142) investigated serum levels of beta-hexosaminidase isoenzymes in relation to risk factors for atherosclerosis in a homogeneous population of postmenopausal women. They found HEX to be associated with several risk factors, such as serum triglycerides, diastolic and systolic blood pressure, glycaemia, waist-to-hip ratio, and body mass index, but not with serum cholesterol. Furthermore, the mean values of HEX-A and HEX-B activity were higher in smokers than in non-smokers, but only the mean activity for HEX-A differed significantly. In addition, the authors demonstrated a correlation between serum HEX and risk factors for myocardial infarction and stroke. In the case of HEX-A, individuals in the 95th–100th percentile of enzyme activity showed a significantly increased risk of myocardial infarction if their fathers had this condition, as well as an increased likelihood of stroke if their mother had it. In the case of HEX-B, individuals in the 95th–100th percentile of enzyme activity showed an increased incidence of stroke if their mothers had a stroke.  Chojnowska et al. (143) analysed correlations between type 2 diabetes (T2D) and serum HEX activity with fat metabolism parameters and symptoms of anxiety and depression. In patients with T2D, a significant positive correlation was found between serum HEX activity and serum concentrations of LDL cholesterol, triacylglycerols (TAGs) and Castelligro atherogenic indexes. A significantly increased level of anxiety and depression was also observed in comparison with the control group. | These studies suggest an association between HEX isoenzymes and the heritability of vascular disease. Since HEX-B is a sensitive marker of AUD, the authors also examined its serum levels in individuals suspected of AUD. However, the study found no differences in these individuals compared to others, probably due to the relatively short half-life of HEX-B after quitting drinking.  Serum HEX activity in patients with T2D is a better marker of atherosclerosis than total serum cholesterol concentration in individuals with mild symptoms of depression and anxiety. Routine screening for anxiety and depression is recommended in patients with T2D. Early detection of these disorders provides an opportunity for treatment, improving the quality of life and overall life expectancy of patients with T2D. |
| Metabolic diseases | | |
| Diabetes | Zalewska-Szajda et al. (150) demonstrated that HEX activity in the saliva of children with type 1 diabetes was significantly higher compared to HEX activity in the saliva of healthy children (p < 0.001). In the group of children with diabetes, HEX activity in saliva was from ~112.19 to ~1264.79 pKat/mL, average 494.33 ± 237.26 pKat/mL; specific HEX activity: from ~0.1360 to ~1.7526 pKat/µg protein, average 0.6266 ± 0.3561 pKat/µg protein. In the control group, HEX activity was from ~126.12 to ~398.40 pKat/mL, average 287.06 ± 111.34 pKat/mL; specific activity was from ~0.1113 to ~0.9908 pKat/µg protein, average 0.40340 ± 0.1978 pKat/µg protein.  The differences between the groups were highly statistically significant: HEX activity concentration: p < 0.00001, specific activity: p < 0.001.  Miller et al. (152) report that GLU activity is significantly higher in the serum of patients with type 1 diabetes compared to the control group. The average enzyme activity was 2,160 units, while in the control group it was 1,260 units.. Increased serum β-glucuronidase activity may be associated with pathological processes occurring in patients with type 1 diabetes.  Severini et al. (151) studied NAG isoenzymes in the serum and urine of patients with diabetes. They showed significantly higher HEX activity in the serum and urine of those patients. Total HEX activity was significantly increased (p < 0.001) in the serum and urine of patients with diabetes and vascular complications, but not in those with diabetes without vascular complications. Isoenzyme A was the predominant form in both serum and urine. Isoenzyme B was present in lower concentrations in the serum of patients with diabetes compared to the control group, and in higher concentrations in the urine of patients with diabetes compared to the control group.  Poon et al. (153) demonstrated a positive correlation between plasma HEX activity and blood glucose concentration in patients with type 1 diabetes treated with insulin.^126^ HEX also showed little diurnal variability, with the lowest level at 7.00 am (p < 0.001). Mean plasma glucose and HEX concentrations fluctuated similarly (r = 0.88, p < 0.01). HEX correlated with changes in plasma glucose and glycated haemoglobin levels (r = 0.61, p < 0.025; r = 0.48, p < 0.05, respectively). | In children with type 1 diabetes, HEX activity in saliva is significantly elevated, both in terms of concentration (pKat/mL) and specific activity (pKat/µg protein), compared to healthy children.  This indicates increased catabolism of glycoconjugates in the saliva of these children, i.e. increased degradation of glycoproteins, glycolipids and proteoglycans. Potentially, HEX activity in saliva could be considered a diagnostic marker or indicator of pathological processes in children with type 1 diabetes, provided that the results are confirmed in larger cohorts.  These results suggest that β-glucuronidase activity may serve as an indicator of disease activity or the extent of tissue damage in diabetes. This can be explained by the intensification of the glucuronic acid cycle, i.e. the glucose metabolism pathway that is insensitive to insulin. This pathway is more active in diabetes, stimulating the synthesis of mucopolysaccharides and glycoproteins, which results in increased GLU activity.  Increased NAG activity in the serum and urine of patients with type 1 diabetes with vascular complications may indicate kidney injury. The observed changes in the ratio of isoenzymes A and B may reflect different pathophysiological mechanisms of diabetes. NAG isoenzyme analysis may be an additional tool in assessing kidney injury in patients with type 1 diabetes.  Plasma HEX activity may be dependent on blood glucose levels in diabetes. |
| Cancers | | |
| Colorectal cancer | Waszkiewicz et al. (160) investigated GLU activity in the blood serum of patients with colorectal cancer (CRC) as a potential diagnostic marker. Patients with colorectal cancer had significantly higher serum GLU activity compared to the control group (p < 0.0001). Serum GLU activity showed high sensitivity (80%) and specificity (82.35%) at a diagnostic threshold of >208.10 pKat/mL. AUC of the diagnostic test was 0.8956 (95% CI: 0.798–0.993), indicating a good potential to differentiate patients with colorectal cancer from healthy individuals.  Szajda et al. (161) examined the activity of HEX and its isoenzymes in the serum and urine of patients with colorectal adenocarcinoma, mucinous colorectal adenocarcinoma and healthy individuals. A significant increase in HEX, HEX-A and HEX-B activity was demonstrated in the serum and urine of patients with colorectal adenocarcinoma compared to healthy individuals. Patients with mucinous colorectal adenocarcinoma presented significantly higher urine HEX activity and significantly higher serum HEX and HEX-B activity expressed as pKat/mg creatinine compared to healthy individuals. A significant increase in HEX, HEX-A and HEX-B activity expressed in pKat/mg creatinine was observed in the urine of patients with tumours measuring 6–7 cm in diameter compared to patients with tumours of 4–5 cm in diameter.  Szajda et al. (131) studied the activity of lysosomal exoglycosidases in the serum and urine of patients with colorectal cancer. They found significantly higher HEX activity in the serum and urine of patients with colorectal cancer compared to controls. GAL activity was also higher in the serum and urine of patients with colorectal cancer compared to the control group. FUC and MAN activity were elevated in the serum, but no significant differences were found in the urine of patients with colorectal cancer compared to controls. | Serum GLU activity may be a promising marker for the diagnosis of colorectal cancer. The results suggest that measuring GLU activity may be helpful in clinical practice.  Determining HEX, HEX-A and HEX-B activity in blood serum and urine can be used to detect early-stage colorectal cancer.  Elevated HEX and GAL activity in the serum and urine of patients with colorectal cancer may indicate tumour cell damage and the release of these enzymes into body fluids. Measuring HEX and GAL activity in serum and urine may be a promising marker in the diagnosis of colorectal cancer. |
| Liver cancer | Gan et al. (164) revealed that the sensitivity of FUC in diagnosing hepatocellular carcinoma (HCC) is 0.72 (95% confidence interval (CI) of 0.69–0.76), with a specificity of 0.78 (95% CI of 0.74–0.81), a diagnostic odds ratio (DOR) of 10.26 (95% CI of 5.99–17.59) and an area under the curve (AUC) of 0.8125.  Wang et al. (165) evaluated the prognostic value of preoperative serum FUC levels in patients with intrahepatic cholangiocarcinoma after liver resection. The authors report that preoperatively high serum FUC activity is a negative predictor of HCC. It was demonstrated that patients with preoperative plasma FUC above 35 uL^-1^ had a significantly lower recurrence-free survival, lower survival rate, and a greater tendency to develop macrovascular invasion. | It was found that FUC offers good diagnostic value in detecting hepatocellular carcinoma, although there are significant population and methodological differences across studies.  Preoperative serum FUC level is an independent prognostic factor in HCC after liver resection. The prognostic value of FUC is significant even in patients with low alpha-fetoprotein (AFP) levels. |
| Pancreatic cancer | Szajda et al. (132) investigated HEX and its isoenzymes (HEX-A and HEX-B) in serum and urine as potential markers of pancreatic cancer. HEX and HEX-A specific activity was significantly higher in the serum of patients with pancreatic adenocarcinoma compared to healthy individuals. However, HEX-B-specific activity in serum and urine did not differ significantly between patients with pancreatic adenocarcinoma and controls. | The results suggest that the activity of HEX and its isoenzyme A, as determined in serum and urine, can be used as a potential marker for pancreatic adenocarcinoma. |
| Kidney cancer | Borzym-Kluczyk et al. (136) reported in their studies that the activity of HEX and its isoenzyme HEX-A was significantly lower in kidney cancer tissue compared to healthy tissue, while in transitional tissue, the enzyme activity was moderate. At the same time, a significant increase in HEX activity was observed in the serum and urine of patients with kidney cancer compared to healthy individuals.  Borzym-Kluczyk et al. (166) studied the activity of HEX isoenzymes A and B in renal tumour tissue. Significantly higher HEX activity was found in renal tumour tissue compared to controls. The activity of isoenzymes A and B was also elevated in renal tumour tissue. These observations confirm that exglycosidases are typical intracellular enzymes released as a result of cell breakdown and cell membrane damage. | In renal tumour tissues, reduced activity of HEX and its isoenzymes is observed compared to healthy tissue, which the authors interpret as a potential effect of changes in tissue composition, degradation of oligosaccharides, proteoglycans, and glycoproteins within cell membranes, the glycocalyx and the extracellular matrix. On the other hand, in the serum and urine of patients with kidney cancer, the activity of HEX and its isoenzymes is increased, which may reflect the release of the enzyme from cancer cells or diseased tissues, or the result of altered secretion, damage or passage through tissue barriers.  The authors suggest that measuring HEX and isoenzymes in serum/urine may offer potential as diagnostic or monitoring markers for kidney cancer.  The increased activity of HEX and its isoenzymes A and B in renal cancer tissue may suggest their role in neoplastic processes and their potential use as diagnostic markers. |
| Thyroid cancer | Zwierz et al. (167) assessed the concentration of thyroid-stimulating hormone (TSH) and the activity of HEX and its isoenzymes A and B in the blood serum of patients with thyroid cancer. They found a significant increase in the activity (p < 0.0191) and specific activity (p < 0.0393) of HEX-A, as well as a decrease in the specific activity of HEX-B, in the serum of patients with thyroid cancer. Normal TSH levels in the blood serum of patients with thyroid cancer confirm euthyroidism in the preoperative period. Determination of the activity and specific activity of HEX-A indicates that, despite euthyroidism, proteins indicative of a pathological process are released into the bloodstream in increased amounts. | Measuring TSH can help confirm euthyroidism in patients with thyroid cancer before surgery. Measuring HEX-A activity may aid in diagnosing thyroid cancer. |
| Brain tumours | Wielgat et al. (169) found increased activity of HEX, HEX-A, HEX-B, GAL and MAN in brain glioma tissues compared to healthy brain tissue and non-glioma tumour tissue. The highest exoglycosidase activity was observed in highly malignant gliomas, specifically glioblastoma multiforme (grade IV), anaplastic astrocytoma (grade III), and anaplastic oligodendroglioma (grade III). HEX and HEX-B activity was six times higher than in normal brain tissue. Exoglycosidase activity in infiltrating tumours was significantly higher than in solid tumours. Furthermore, a positive correlation was observed between the activity of HEX, HEX-A and HEX-B and the malignancy grade of primary brain tumours. | These results suggest that lysosomal exoglycosidases may be involved in the progression and dynamic development of gliomas. |
| Salivary gland tumours | Borzym-Kluczyk et al. (170) report that the activity of HEX and its isoenzymes HEX-A and HEX-B in the tissue of pleomorphic adenoma, a benign tumour of the salivary gland, is significantly elevated. In salivary gland tumour tissue, the activity of HEX, HEX-A, HEX-B, GAL and FUC was considerably higher compared to healthy tissue. The activity of all examined lysosomal exoglycosidases (HEX, HEX-A, HEX-B, GLU, GAL, MAN and FUC) in the serum of patients with salivary gland tumours was significantly higher compared to the serum of healthy controls. The saliva of patients with salivary gland tumours also showed significantly higher activity of HEX, HEX-A, GLU, GAL, MAN, and FUC, and a downward trend in HEX-B activity compared to that of healthy controls. | The assessment of serum and saliva HEX levels in patients with salivary gland tumours could be potentially employed in diagnosing and monitoring salivary gland tumours. |
| Laryngeal cancer | Olszewska et al. (171) attempted to evaluate MAN and GAL activity in squamous cell carcinoma of the larynx. The authors observed higher MAN activity in all laryngeal cancer samples compared to healthy tissue samples. The mean MAN activity was 3.702 ± 1.3245 nkat/g wet tissue compared to the control group (1.614 ± 0.8220 nkat/g wet tissue). The authors also showed significantly higher GAL activity in laryngeal cancer compared to healthy tissue samples. The mean GAL activity was 3.383 ± 2.1980 nkat/g wet tissue compared to the control group (2.137 ± 1.3685 nkat/g wet tissue). | The activity of MAN and GAL is significantly elevated in laryngeal cancer compared to healthy tissue, indicating that catabolic reactions involving glycoproteins, glycolipids and proteoglycans may play a role in the pathogenesis of laryngeal cancer. |
| Other tumours | Severini et al. (172) determined HEX and GLU activity in the serum of patients with breast and gastrointestinal cancer and in healthy individuals. HEX levels elevated above the normal range (45.6 U/L) were observed in 78% patients with breast cancer, 100% with gastric cancer, 70% with colorectal cancer, 100% with liver cancer, and 100% with pancreatic cancer. In patients with breast and gastric cancer, HEX showed good specificity (90% and 91%, respectively) and sufficient sensitivity (78% and 100%, respectively) as a potential tumour marker. GLU proved less sensitive and was significantly elevated (in all patients) only in the case of pancreatic cancer. | The authors suggest that NAG may be a valuable marker for diagnosing certain types of cancer, particularly those involving the liver and pancreas, as its levels are significantly elevated in those patients. However, the low specificity limits its applicability as a standalone marker—an increase in HEX alone is not sufficient to conclude that a patient has cancer, as it may also be elevated for other reasons. |
| Nasopharyngeal diseases | | |
| Nasal polyps | Chojnowska et al. (178) observed significantly lower GAL and FUC activity, as well as a trend towards reduced MAN and GLU expression, in nasal polyp tissue compared to healthy mucosa. For MAN and GLU, a downward trend was observed, but the differences were not statistically significant. When enzyme activity was expressed as specific activity, no significant differences were found between polyps and control tissue for GAL, MAN and FUC. | The findings suggest that nasal polyps show reduced activity of certain lysosomal exoglycosidases (particularly GAL and FUC) compared to healthy mucosa.  The authors explain this by deregulation of inflammatory mechanisms. |
| Chronic tonsillitis | Popko et al. (186) examined the activity and specific activity of HEX, HEX-A, HEX-B, GLU, GAL, MAN and FUC in both hypertrophied and inflamed palatine tonsil tissue (in children and adults). The activity and specific activity of each of the examined lysosomal exoglycosidases were significantly higher in each of the three pathological tissue groups compared to healthy tonsil tissue. No significant differences in the inflamed tonsils were found based on age. | This suggests that complete removal of the palatine tonsils (tonsillectomy) is justified in cases of both hypertrophy and inflammation (in childhood). |
| Rheumatic and musculoskeletal diseases | | |
| Inflammatory joint diseases | Pancewicz et al. (193) showed that HEX, GAL and MAN activity were significantly higher in the serum of patients with Lyme arthritis compared to healthy individuals and normalised after treatment with doxycycline. HEX activity in the serum of patients with Lyme arthritis was significantly higher compared to controls (p < 0,001). After 4 weeks of treatment with doxycycline, this activity tended to decrease, but was still higher than in healthy individuals. GAL and MAN activity in patients with Lyme arthritis before therapy was elevated compared to the control group; after treatment, it fell to almost the same level as that observed in healthy individuals. These changes were not statistically significant, but the trend was consistent.  Pancewicz et al. (130) compared the activity of HEX, GLU, GAL, MAN and FUC in the serum and synovial fluid of patients with Lyme disease, rheumatoid arthritis and juvenile idiopathic arthritis. A significant increase in the activity of all exoglycosidases in serum and synovial fluid was observed in patients with various forms of arthritis compared to controls. The ratio of exoglycosidase activity in synovial fluid to exoglycosidase activity in serum was above 2 in patients with Lyme disease, but not in patients with rheumatoid arthritis or juvenile idiopathic arthritis. | Studies suggest that HEX may be a sensitive enzymatic marker for monitoring disease progression and treatment efficacy in Lyme arthritis.  Since the synovial membrane is the primary source of exoglycosidases in the joint, this finding supports the therapeutic value of synovectomy in chronic Lyme arthritis with knee effusion. Serum HEX activity assessment can be used to monitor the course of Lyme arthritis and the efficacy of treatment. |
| Systemic sclerosis | Knaś et al. (196) found significantly higher HEX, HEX-A, HEX-B and GLU activity in the unstimulated saliva of patients with systemic sclerosis compared to the control group. A negative correlation was also demonstrated between HEX-B activity and unstimulated saliva secretion per minute, suggesting that disorders of glycoconjugate catabolism contribute to salivary hypofunction in patients with systemic sclerosis. | GLU is secreted mainly by inflammatory cells (neutrophils, macrophages), which means that increased enzyme activity in saliva may be an indicator of early, clinically asymptomatic damage to the parotid glands in the course of systemic sclerosis. |
